# Mining and engineering exporters for titer improvement of macrolide biopesticides in *Streptomyces*

**DOI:** 10.1111/1751-7915.13883

**Published:** 2021-08-26

**Authors:** Liyang Chu, Shanshan Li, Zhuoxu Dong, Yanyan Zhang, Pinjiao Jin, Lan Ye, Xiangjing Wang, Wensheng Xiang

**Affiliations:** ^1^ School of Life Science Northeast Agricultural University No. 59 Mucai Street, Xiangfang District Harbin 150030 China; ^2^ State Key Laboratory for Biology of Plant Diseases and Insect Pests Institute of Plant Protection Chinese Academy of Agricultural Sciences Beijing 100193 China

## Abstract

Exporter engineering is a promising strategy to construct high‐yield *Streptomyces* for natural product pharmaceuticals in industrial biotechnology. However, available exporters are scarce, due to the limited knowledge of bacterial transporters. Here, we built a workflow for exporter mining and devised a tunable plug‐and‐play exporter (TuPPE) module to improve the production of macrolide biopesticides in *Streptomyces*. Combining genome analyses and experimental confirmations, we found three ATP‐binding cassette transporters that contribute to milbemycin production in *Streptomyces bingchenggensis*. We then optimized the expression level of target exporters for milbemycin titer optimization by designing a TuPPE module with replaceable promoters and ribosome binding sites. Finally, broader applications of the TuPPE module were implemented in industrial *S. bingchenggensis* BC04, *Streptomyces avermitilis* NEAU12 and *Streptomyces cyaneogriseus* NMWT1, which led to optimal titer improvement of milbemycin A3/A4, avermectin B_1a_ and nemadectin α by 24.2%, 53.0% and 41.0%, respectively. Our work provides useful exporters and a convenient TuPPE module for titer improvement of macrolide biopesticides in *Streptomyces*. More importantly, the feasible exporter mining workflow developed here might shed light on widespread applications of exporter engineering in *Streptomyces* to boost the production of other secondary metabolites.

## Graphical abstract

We developed a workflow to enable the genome‐wide mining of latent exporters and designed a tunable plug‐and‐play exporter module using these exporters to boost titers of different macrolide biopesticides. Our work not only provides useful exporters contributing to macrolide biopesticide production, but also presents a paradigm for exporter exploration and application in other *Streptomyces*.

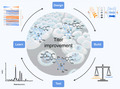

## Introduction

Natural products produced by *Streptomyces* species play crucial roles in clinical and agricultural fields (Liu *et al*., 2013). Titer improvement of these pharmaceuticals is a perennial subject of interest. Great advances have thus far been achieved through various sophisticated strategies, such as precursor engineering (Lu *et al*., 2016; Li *et al*., 2021), regulatory engineering (Martin and Liras, 2010; Liu *et al*., 2013), biosynthetic gene cluster (BGC) manipulation (Domrose *et al*., 2017; Li *et al*., 2017), and omics‐guided and model‐based system engineering (Dias *et al*., 2015; Horinouchi *et al*., 2017). While admittedly, overaccumulation of the target compounds within cells often results in tolerance burden and restrains the final production levels (Mukhopadhyay, 2015; Naseri and Koffas, 2020). Therefore, strategies addressing this issue should also be fully considered when engineering the high‐yield *Streptomyces*.

Exporters as the door of product output are undoubtedly significant tools to cope with this issue. Exporter engineering has been successfully applied to enhance production of organic acids, amino acids and biofuels in *Escherichia coli*, *Bacillus subtilis* and yeast (Fisher *et al*., 2014; Jones *et al*., 2015; Averesch *et al*., 2018; Zhu *et al*., 2020). By contrast, applications of this approach are rare in *Streptomyces*. Almost all investigated drug exporters of *Streptomyces* are involved in corresponding BGCs (named as BGC‐linked exporters) (Severi and Thomas, 2019). The transcriptional levels of some BGC‐linked exporters are bound up with the titer of corresponding products, such as ActAB of *Streptomyces coelicolor* and DrrAB of *Streptomyces peucetius* (Srinivasan *et al*., 2010; Xu *et al*., 2012). While this is not always the case, for instance, overexpression of *lndJ* encoding an exporter of landomycin E resulted in abolishment of production rather than enhancement in *Streptomyces globisporus* (Ostash *et al*., 2007). Moreover, some BGCs were lack of exporter encoding genes, such as the milbemycin BGC in *Streptomyces bingchenggensis* (Zhang *et al*., 2016), and some exporters that are located far from target BGCs could also facilitate antibiotic efflux, such as Mfs1 and NepI/II for natamycin exportation (Wang *et al*., 2017). Given that *Streptomyces* genomes are rich in exporters (Saidijam *et al*., 2006; Zhou *et al*., 2016), it is quite possible to obtain valuable exporters for promotion of exporter engineering in this species.

Functional mining of exporters has up to now been a challenging issue (Wang *et al*., 2021). Commonly used approaches are mainly sequence‐dependent methods, while it is difficult to generate accurate predictions, due to the extremely complex relationship between transporter sequence and function (Genee *et al*., 2016). Recently, biosensor‐based strategies have been developed for transporter identification (Genee *et al*., 2016; Wang *et al*., 2021). Based on fluorescence‐activated cell sorting or dual‐selection on agar plates, these high‐throughput strategies advanced transporter exploration and function analysis in *E. coli* and *Saccharomyces cerevisiae*. Nevertheless, applications of these strategies are restrained in *Streptomyces* because of its complex physiological and morphological characteristics. Therefore, development of an exporter mining method is necessary to facilitate strain engineering of *Streptomyces*.

The macrolide natural products, such as avermectins, milbemycins and their derivatives, are valuable biopesticides in the agricultural field (William, 2012; Arsic *et al*., 2018). Producers of these compounds, especially promising milbemycins, urgently require strain improvements to ensure high yield and low production cost (Martin *et al*., 2012; Jacobs and Scholtz, 2015; Rinaldi *et al*., 2015). Therefore, in our research, exporter mining was first carried out in *S*. *bingchenggensis*, a well‐investigated milbemycin producer originally isolated by our laboratory (Xiang *et al*., 2007; Wang *et al*., 2010; Zhang *et al*., 2016; Jin *et al*., 2020). Here, we developed a workflow to enable genome‐wide mining of latent exporters contributing to milbemycin production and further designed a tunable plug‐and‐play exporter (TuPPE) module using these exporters to improve the titer of different macrolide biopesticides in their corresponding producers. Our work provided useful exporters for titer improvement of macrolide biopesticides in *Streptomyces*. Moreover, our exporter mining workflow might be applicable to explore other interesting transporters for titer improvement of desired products in *Streptomyces*.

## Results

### Milbemycin production is influenced by exporters in *S. bingchenggensis*

**Fig. 1:**
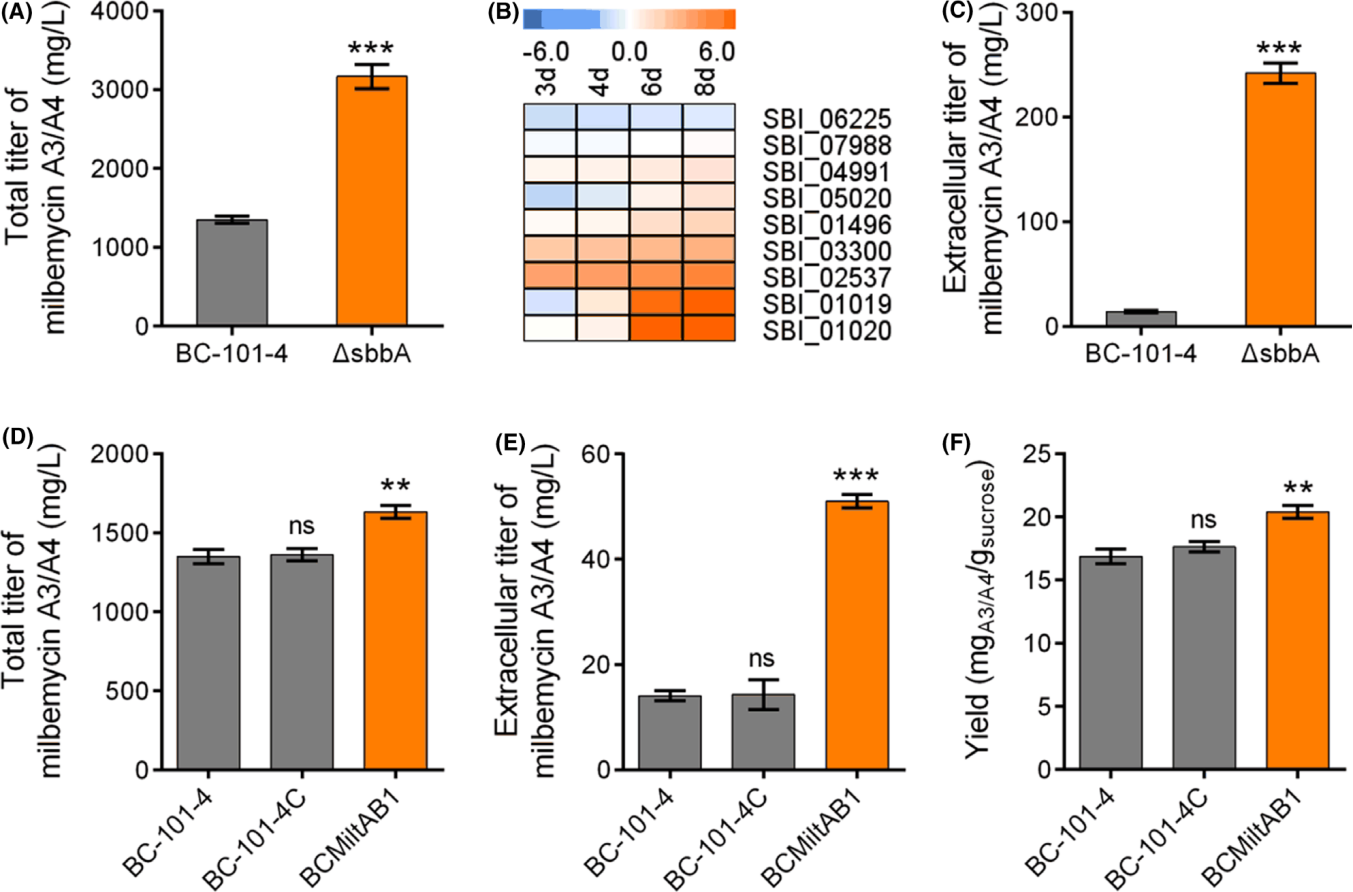
Discovering and confirmation of MiltAB1 contributing to milbemycin production in *S. bingchenggensis*. A. Comparison of total milbemycin production between BC‐101‐4 and ΔsbbA. BC‐101‐4, the parent strain; ΔsbbA, a high‐yield milbemycin producer. B. Transcription profile of nine genes correlated with the titer of milbemycin in ΔsbbA. C. Comparison of extracellular milbemycin production between BC‐101‐4 and the mutant ΔsbbA. D. and E. Comparison of total and extracellular milbemycin production between engineered strains and their parent strain BC‐101‐4. BC‐101‐4C and BC‐101‐4 integrated with the control plasmid (pSET152T); BCMiltAB1 was the strain overexpressing the MiltAB1 in BC‐101‐4. F. Comparison of yield between engineered strains and their parent strain BC‐101‐4. For (A and C–F), the data of BC‐101‐4 were set as control, differences were analyzed by Student’s *t*‐test, and *P* < 0.05 was considered statistically significant. The levels of significance are ***P < 0.001, **P < 0.01 and *P < 0.05 and ‘ns’ means no significant difference. Data shown were the averages and standard deviations. All data were obtained from three independent experiments.

The high‐yield milbemycin strain ΔsbbA was obtained in our previous work (He *et al*., 2018). It showed a 2.3‐fold increase of milbemycin A3/A4 titer compared to that of the parent BC‐101‐4 (Fig. 1A). To distinguish which genes contributed to the titer improvement, we analyzed the transcriptomic data obtained from the main production stage (3–8 day) of ΔsbbA and got 79 genes with transcriptional levels that were closely correlated with milbemycin production (*r* ^2^ > 0.995), including 17 genes with transcriptional levels that were significantly changed (slope > 0.05) (Dataset S1). Meanwhile, we chose genes with relatively higher transcriptional levels at day eight (log_10_^FPKM^ > 1) (Fig. S1) and finally obtained nine genes with defined annotations (Fig. S2). Additionally, we further compared time‐course transcriptional levels of these genes between ΔsbbA and BC‐101‐4. We noted that a group of putative ATP‐binding cassette (ABC) transporter systems (SBI_01019 and SBI_01020, named MiltAB1) exhibited significantly upregulated the transcriptional level at the late production stage (Fig. 1B and Fig. S3). Correspondingly, the extracellular titer of milbemycin A3/A4 of ΔsbbA showed a 17.2‐fold increase compared to that of BC‐101‐4 (Fig. 1C), suggesting MiltAB1 might be an exporter responsible for milbemycin efflux.

We further overexpressed MiltAB1 under the control of the *hrdB* promoter (*hrdB*p) in BC‐101‐4, which led to a 20.9% and 361.7% titer increase of total and extracellular milbemycin A3/A4 (Fig. 1D and E). Moreover, the yield of milbemycin A3/A4 was also improved by 21.4% (Fig. 1F). These data show the important role of MiltAB1 in promoting milbemycin production and further suggest the necessity of mining other latent exporters in the *Streptomyces* genome for high‐yield strain construction.

### In depth mining of exporters contributing to milbemycin biosynthesis

**Fig. 2:**
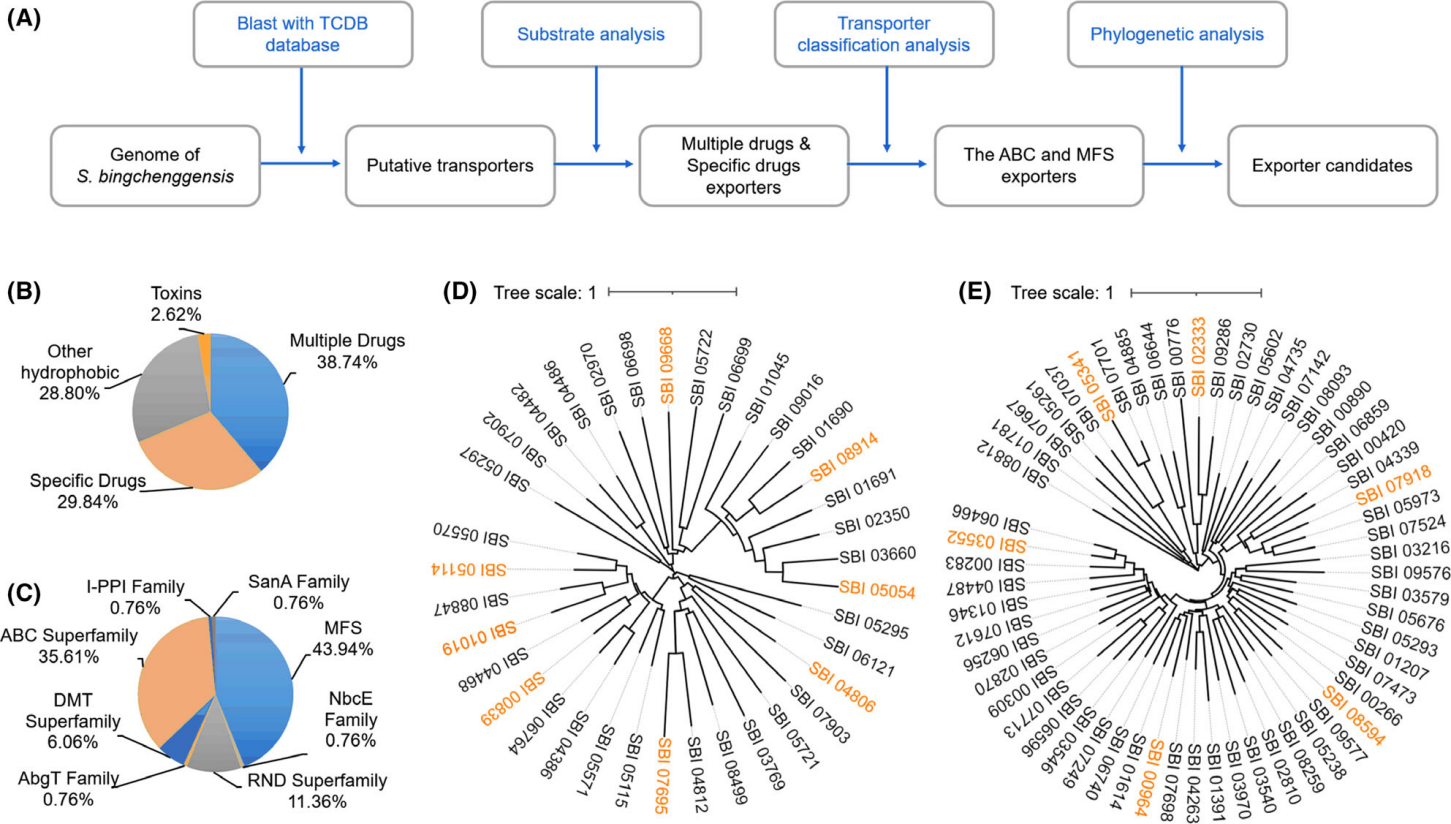
In depth mining of exporters contributing to milbemycin biosynthesis in *S. bingchenggensis*. A. Workflow for exporter systems mining. B. Substrate analysis of the *in silico* selected exporter systems of *S. bingchenggensis*. C. Transporter classification of the putative drug exporter systems. MFS, the major facilitator superfamily; NbcE, the novobiocin exporter family; RND, the resistance‐nodulation‐cell division superfamily; AbgT, the *p*‐aminobenzoyl‐glutamate transporter family; DMT, the drug/metabolite transporter superfamily; ABC, the ATP‐binding cassette superfamily; I‐PPI, the immunophilin‐like prolyl:peptidyl isomerase regulator family; SanA, the vancomycin‐sensitivity protein (SanA) family. D. and E. Phylogenetic tree analysis of ABC exporters (proteins with TMD) and MFS exporters, respectively. The exporters marked as orange were picked out as candidates. SBI_01019 belonging to MiltAB1 was marked as dark orange.

The genomes of *Streptomyces* species contain a plethora of transporters. For example, transporters accounted for 12.1%, 13.7% and 12.9% of the respective proteome of *S. coelicolor*, *Streptomyces griseus* and *Streptomyces avermitilis* (Zhou *et al*., 2016). Notably, the number of transporters in *S. bingchenggensis* was up to 1063 (Jin *et al*., 2020). Since evolution would generally confer the host greater survival advantage, we posited that more than one exporter might exist for milbemycin efflux. To mine more favorable exporters of milbemycins, we performed a step‐by‐step analysis workflow (Fig. 2A) and selected 191 proteins that might be responsible for efflux of drugs, dyes, sterols and toxins. These proteins included 74 putative multiple drug efflux proteins and 57 putative specific drug efflux proteins according to substrate analysis (Fig. 2B and Dataset S2). Transporter classification analysis showed that the 131 proteins included 46 ABC superfamily proteins (belonging to 36 ABC exporter systems) and 58 major facilitator superfamily (MFS) proteins, accounting for 79.4% of the total putative drug efflux proteins (Fig. 2C and Dataset S2).

ABC superfamily exporters of bacteria generally function by dimerizing ‘half‐transporters’, which share a common architecture consisting of one transmembrane domain (TMD) fused to one nucleotide‐binding domain (NBD) (Dawson and Locher, 2006; Boncoeur *et al*., 2012). We manually checked and added 21 putative proteins containing TMD and two putative proteins containing NBD in the 36 ABC exporter systems where TMD or NBD were missing (Dataset S3). Phylogenetic analysis is a useful approach to obtain the desired target (Wang *et al*., 2020). Therefore, we performed phylogenetic analysis of TMDs, which generally exhibit high sequence variability and play an important role in substrate recognition (Dawson and Locher, 2006; Chen *et al*., 2012). Proteins from different branches usually have different characteristics. To obtain desired exporters, in addition to MiltAB1, we selected seven additional candidates (MiltAB2, SBI_00839−SBI_00840; MiltAB3, SBI_07695−SBI_07696; MiltAB4, SBI_04806−SBI_04807; MiltAB5, SBI_05053−SBI_05054; MiltAB6, SBI_05114−SBI_05116; MiltAB7, SBI_08914−SBI_08915; MiltAB8, SBI_09667−SBI_09668) among the 36 analyzed ABC exporter systems belonging to different branches (Fig. 2D and Dataset S3). MFS is also one of the largest groups of secondary active transporters (Yan, 2013). Similarly, we performed phylogenetic analysis of the 58 MFS exporters and chose six (MilMFS1, SBI_02333; MilMFS2, SBI_03552; MilMFS3, SBI_05341; MilMFS4, SBI_08594; MilMFS5, SBI_00964; MilMF6, SBI_07918) from different branches for further experimental validation.

### Evaluation of exporters in *S. bingchenggensis*

**Fig. 3:**
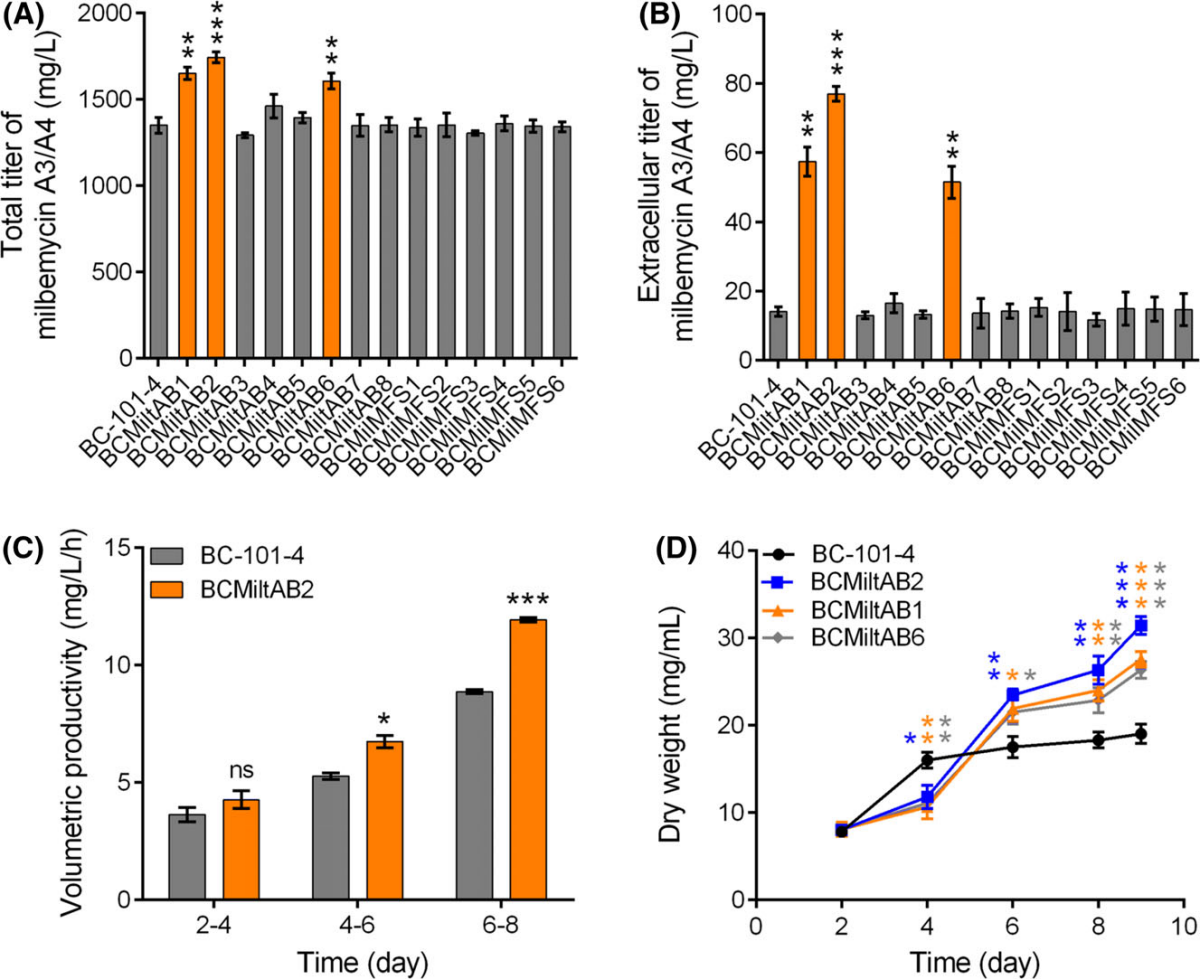
Evaluation of exporters in *S. bingchenggensis*. A. Effect of exporter overexpression on total titer of milbemycin A3/A4. B. Effect of exporter overexpression on extracellular titer of milbemycin A3/A4. C. Productivity comparison between BCMiltAB2 and BC‐101‐4 at different fermentation stages. D. Influence of constitutive overexpression of exporters on cell growth. For (A−D), the data of BC‐101‐4 were set as control. All data were obtained from three independent experiments. Data shown were the averages and standard deviations. Differences were analyzed by Student’s *t*‐test; the levels of significance are ***P < 0.001, **P < 0.01, and *P < 0.05; ‘ns’ means no significant difference.

We individually overexpressed the 14 candidate exporter systems (MiltAB1 to MiltAB8, and MFS1 to MFS6) under the control of the constitutive promoter *hrdB*p in BC‐101‐4. Among all tested candidates, MiltAB1, MiltAB2 and MiltAB6 showed significant contributions to both total titer enhancement and milbemycin efflux (Fig. 3A and B). MiltAB2 was the best performer and resulted in a 29.1% increase of total titer of milbemycin A3/A4, which was 6.9% and 10.1% higher than the effect caused by MiltAB1 and MiltAB6 overexpression, respectively (Fig. 3A). Correspondingly, the extracellular titer of milbemycin A3/A4 in BCMiltAB2 was 5.5 times of that in BC‐101‐4, showing a 1.4‐ and 1.9‐fold increase compared to that of BCMiltAB1 and BCMiltAB6 (Fig. 3B). It was notable that TMD‐involved proteins of these three exporter systems (SBI_01019, SBI_00839 and SBI_05114) were clustered in the same branch, while other tested candidates were at a greater distance and showed little effect (Fig. 2D and E, 3A and B), suggesting proteins in this branch might share similar substrate binding sites and contribute to milbemycin efflux. Considering the high redundancy of transporters (Mans *et al*., 2017), gene deletion to confirm roles of the identified transporters was not performed.

We observed that the milbemycin productivity difference between the parent strain and BCMiltAB2 was gradually enhanced along with the fermentation process (Fig. 3C). Similar trends were also observed in BCMiltAB1 and BCMiltAB6 (Fig. S4). In addition, growth of the three engineered strains were all inhibited at the early stage of fermentations, while this issue was gradually alleviated as time went by (Fig. 3D), indicating metabolic burden resulted from constitutive overexpression of membrane proteins (Naseri and Koffas, 2020). To confirm this point, we chose two promoters (the strong promoter *kasO*p* and the native promoter P*_sbi_00840_* of the MiltAB2) with distinct expression profile from *hrdB*p to overexpress MiltAB2 in BC‐101‐4 (Wang *et al*., 2013), respectively (Fig. S5). Compared to using *hrdB*p, P*_sbi_00840_* could alleviate cell growth inhibition, whereas *kasO*p* let it worse (Fig. S6). These data suggest that it might be quite necessary to handle the trade‐off between the exporter overexpression level and cell growth to avoid metabolic burdens (Xu *et al*., 2020). We therefore designed a tunable module to control the temporal expression level of the target exporters.

### Fine‐tuning exporter expression for titer optimization in *S. bingchenggensis*

**Fig. 4:**
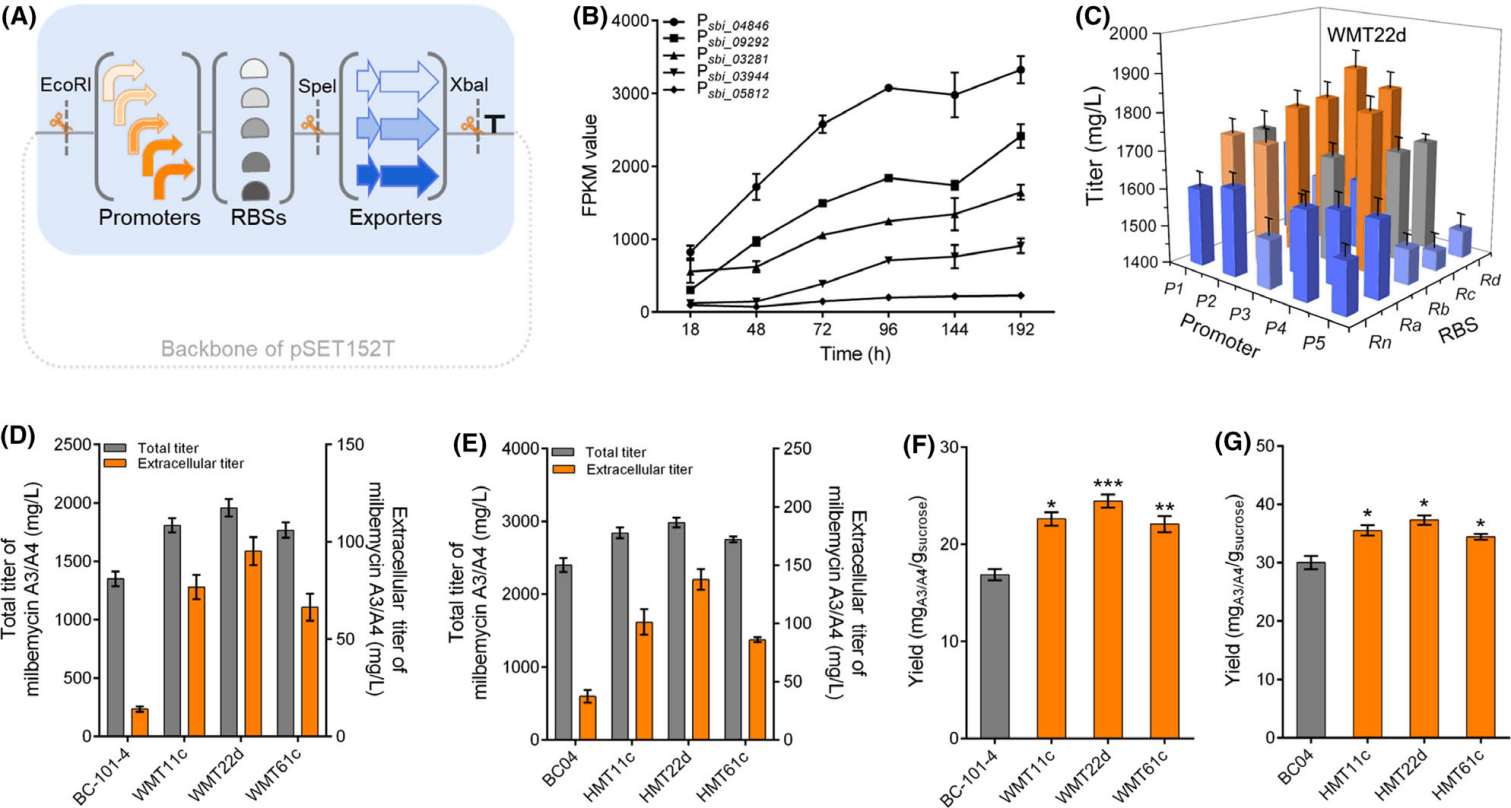
Titer optimization by the TuPPE module. A. Design of the TuPPE module. B. Profile of the five selected promoters in BC‐101‐4. Promoter strength was indicated by gene FPKM values from transcriptome data (GSE166795). C. Influence of the TuPPE module containing MiltAB2 on milbemycin production. D. and E. Production comparison between the optimal engineered strains and their corresponding parent strain BC‐101‐4 and BC04, respectively. F. and G. Comparison of yield between the optimal engineered strains and their corresponding parent strain BC‐101‐4 and BC04, respectively. Strain names containing MT11c, MT22d and MT61c represent the strains integrated with the MiltAB1, MiltAB2 and MiltAB6 involved the TuPPE module in the corresponding host. For (B–G), data shown were the averages and standard deviations and obtained from three independent experiments. For (D) and (F), data of BC‐101‐4 were set as control. For (E) and (G), data of BC04 were set as control. Differences were analyzed by Student’s *t*‐test; the levels of significance are ***P < 0.001, **P < 0.01 and *P < 0.05; ‘ns’ means no significant difference.

To avoid the metabolic burden caused by exporter overexpression, we devised a user friendly, plug‐and‐play TuPPE module. Considering that promoter and ribosome binding site (RBS) are generally used regulatory components controlling transcriptional and translational machinery (Nielsen and Keasling, 2016), we planned to fine‐tune the overexpression levels of target exporters using both of them. Moreover, specific restriction enzyme sites were designed in the TuPPE module to facilitate genetic part assembling (Fig. 4A). To cope with cell growth inhibition during the logarithmic phase, we expected to choose promoters with relatively low strength at the logarithmic phase while significantly upregulated strength at the milbemycin‐producing period. Based on clustering analysis of the time‐course transcriptome data of BC‐101‐4 (GEO No: GSE166795), we first obtained 401 promoters that had satisfactory transcriptional patterns (Fig. S7 and Dataset S4). Then, according to the transcriptional level of each gene, we selected five promoters (P*_sbi_04846_*, P*_sbi_09292_*, P*_sbi_03281_*, P*_sbi_03944_* and P*_sbi_05812_*, named P1 to P5) with different strength gradients (Fig. 4B and C and Table S4). In addition, four RBSs (named Ra to Rd) from commonly used cloning vectors were selected to give a range of exporter expression levels (Fig. 4C and Table S5). For comparison, the native RBS (indicated by R*n*, *n* is from 1 to 5) at the downstream of the five selected promoters were also used.

We paired up the selected promoters and RBSs and generated 5 × 5 combinations (TuPPE*_n_*‐MiltAB2) to control the expression level of MiltAB2 in BC‐101‐4. Strain WMT22d with MiltAB22d (MiltAB2 driven by P2 and Rd) resulted in the highest milbemycin A3/A4 titer of 1958.0 mg l^‐1^ (Fig. 4D), 12.3% higher than BCMiltAB2 using a constitutive strong promoter *hrdB*p to control the expression level of MiltAB2. Likewise, the extracellular titer of A3/A4 was increased by 23.6% (Fig. 4D). As expected, metabolic burden observed in BCMiltAB2 was abolished in WMT22d with a fine‐tuned overexpression level of MiltAB2 (Fig. S6), further suggesting the importance of fine‐tuning the expression of exporters.

The identified MiltAB1 and MiltAB6 were manipulated using the same strategy to construct TuPPE*_n_*‐MiltAB1 and TuPPE*_n_*‐MiltAB6 modules. These modules were also transformed into BC‐101‐4 (Fig. S8). The resulting strains, WMT11c with MiltAB11c (MiltAB1 driven by P1 and Rc) and WMT61c with MiltAB61c (MiltAB6 driven by P1 and Rc), led to production enhancement of 9.5% and 10.0% to the highest titer of 1807.0 mg l and 1767.0 mg l^‐1^, respectively (Fig. 4D). Similarly, extracellular titer increased by 33.7% and 28.9%, respectively, compared to the corresponding strains utilizing the *hrdB*p promoter (Fig. 4D).

To test the effect of the TuPPE module in high‐yield strains, we further individually integrated TuPPE modules MiltAB22d, MiltAB11c and MiltAB61c into the high‐yield industrial producing strain BC04. The resulting strain HMT22d, HMT11c, and HMT61c improved the total production by 24.2%,18.4% and 14.7%, respectively, compared to BC04. Extracellular production was also increased by 3.7, 2.7 and 2.3 times (Fig. 4E). In addition, the yield of milbemycin A3/A4 was improved by 44.4% and 24.3% in WMT22d (24.4 mg_A3/A4_/g_sucrose_) and HMT22d (37.3 mg_A3/A4_/g_sucrose_) compared to their individual parent strain (Fig. 4F and G). These data suggest the great potential of these previously unidentified exporters and emphasized the necessity of exporter fine‐tuning for milbemycin titer and yield improvement in *S. bingchenggensis*.

### Titer improvement of different macrolide biopesticides by exporter engineering

**Fig. 5:**
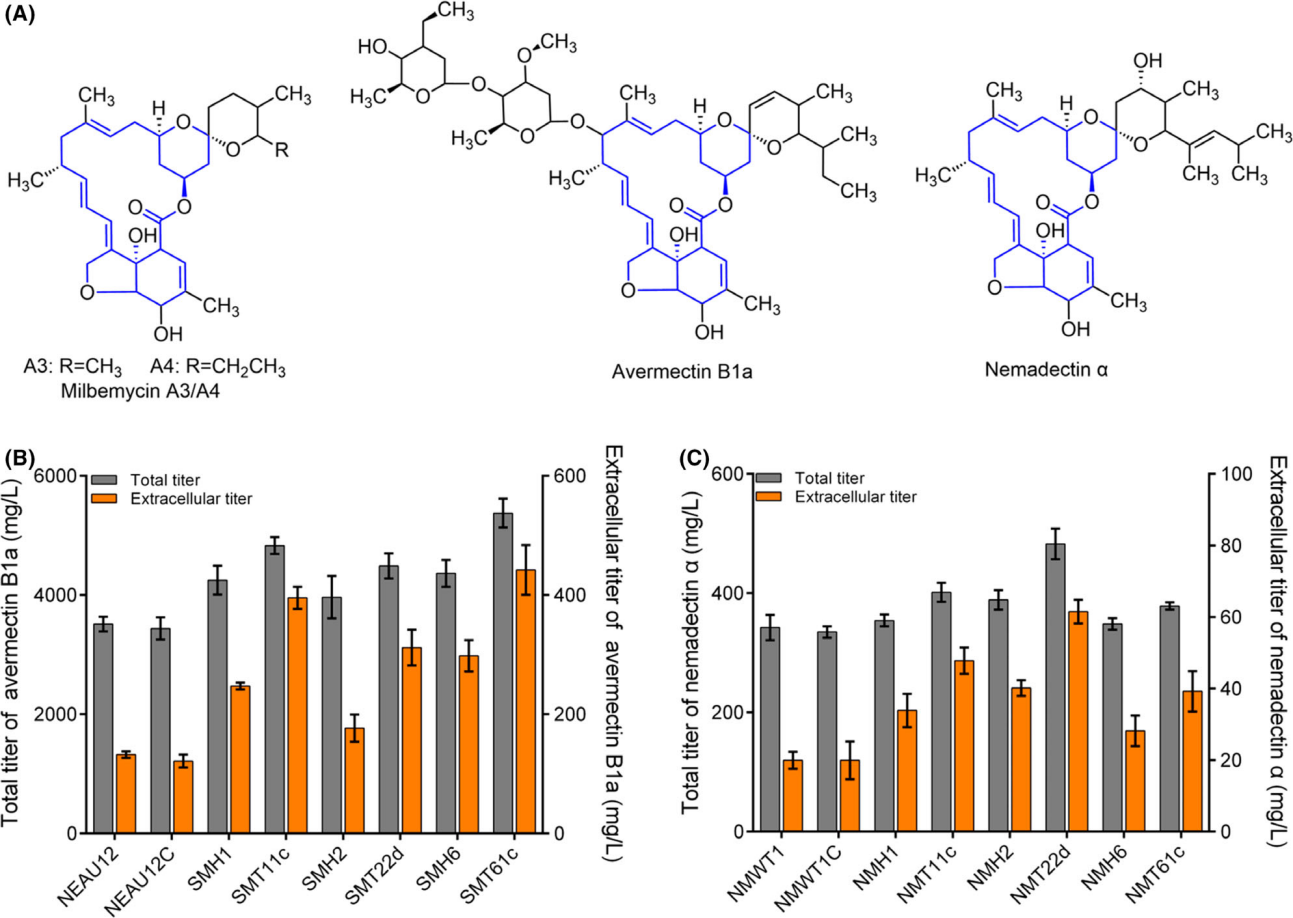
Broader application of the TuPPE module for titer improvement of different macrolide biopesticides. A. Comparison of polyketide skeletons of different 16‐membered macrolide antibiotics. The blue part indicates the polyketide skeleton. B. Performance of overexpression of MiltAB1, MiltAB2 and MiltAB6 on production of avermectin B_1a_. C. Performance of overexpression of MiltAB1, MiltAB2 and MiltAB6 on production of nemadectin α. Strain names containing MH1, MH2 and MH6 represent the strains integrated with MiltAB1, MiltAB2 and MiltAB6, respectively, controlled by *hrdB*p in the corresponding hosts. Strain names containing MT11c, MT22d and MT61c represent the strains integrated with MiltAB1, MiltAB2 and MiltAB6 involved TuPPE modules in the corresponding hosts, respectively. For (B) and (C), Data shown were the averages and standard deviations and obtained from three ndependent experiments.

Considering the similarities of polyketide skeletons and production patterns of macrolide biopesticides such as milbemycins, avermectins and nemadectin (Fig. 5A and Fig. S9), we speculated that the three TuPPE modules, MiltAB11c, MiltAB22d and MiltAB61c, might be favorable for the other two natural products besides milbemycins. To confirm our speculations, these TuPPE modules were introduced into the avermectin producer *S. avermitilis* NEAU12 and nemadectin producer *Streptomyces cyaneogriseus* NMWT1, respectively. In comparison to NEAU12, the titer of avermectin B_1a_ was enhanced by 38.4% (4829.7 mg l^‐1^), 27.8% (4488.3 mg l^‐1^) and 53.0% (5373.5 mg l^‐1^) in SMT11c, SMT22d and SMT61c, respectively (Fig. 5B). Consistent with those results, the extracellular titer was increased by 3.0‐, 2.4‐ and 3.3‐fold, respectively (Fig. 5B). In *S. cyaneogriseus* NMWT1, the MiltAB22d TuPPE module resulted in a 41.0% titer improvement of nemadectin α to 482.6 mg l^‐1^, while MiltAB11c and MiltAB61c led to 17.3% and 10.6% titer increases, respectively. These results were consistent with the extracellular production which increased by 3.1, 2.4, and 2.0 times, respectively (Fig. 5C). The yields of avermectin B_1a_ and nemadectin α reached maximum improvements of 53.0% and 42.6% in SMT61c (38.4 mg_avermectin B1a_/g_starch_) and NMT22d (19.3 mg_nemadectin α_/g_lactose_) over their individual controls (Fig. S10). Productions of engineered strains using TuPPE modules were much higher than those using the strong constitutive *hrdB*p controlled exporter overexpression module (Fig. 5B and C). In addition, cell growth inhibition during the logarithmic phase was not observed in all TuPPE employed engineering strains (Fig. S11). These results demonstrated that the three transporters have evolved to play a promiscuous role of macrolide efflux, and their manipulation of them could efficiently improve the titer of avermectin B_1a_ and nemadectin α, although the efficiency of a given TuPPE module may vary in different *Streptomyces*. Nevertheless, fine‐tuning the expression of exporters was necessary for high‐yield strain engineering.

## Discussion

Exporter engineering has been proven to be a significant strategy to improve transmembrane transfer efficiency, protect cells from toxic products, and enhance microbial production in various bacteria (Lv *et al*., 2016; Steiger *et al*., 2019). Application of such a strategy has been rare for titer improvement of desired chemicals in *Streptomyces*, the well‐known industrial producer of clinically and agriculturally used natural products (Liu *et al*., 2013). In the past decades, knowledge of the role of transporter systems in the secretion of secondary metabolites has advanced, but for the vast majority their functions were remain unclear. For example, the number of transporters without clearly functional annotations is up to 857 in *S. bingchenggensis*, accounting for 80.6% of all transporters (Jin *et al*., 2020). Existing exporter research mainly focused on the BGC‐linked efflux systems in *Streptomyces* due to the high flexibility of transporter substrate specificity (Martín *et al*., 2005; Genee *et al*., 2016; Severi and Thomas, 2019). Since *Streptomyces* have a large number of unidentified transporters, exporter discovery and application is a promising strategy for high‐yield strain construction.

Genome sequence analysis reveals the presence of numerous drug transporters in *Streptomyces* (Zhou et al., 2016), whereas the number of drug transporters is much lower (4–8 per genome) in *E. coli*, *Bacillus subtilis* and other actinomycetes, such as *Mycobacterium tuberculosis* or *Corynebacterium diphtheriae* (Saidijam *et al*., 2006). Given that *Streptomyces* species produce much more secondary metabolites than other species, we speculated that there might be contributing exporters that are located far from BGCs (called BGC‐independent exporter). The following facts might also support our assumption: first, many multidrug exporters in *Streptomyces* could expel different secondary metabolites with a related structure to protect hosts during evolution (Yazaki, 2005); second, target secondary metabolites can be pumped out in the absence of BGC‐linked exporters (Zhang *et al*., 2016), such as milbemycins produced by *S. bingchenggensis* (Fig. 1); third, deletion of some BGC‐linked exporters like AvtAB in *S. avermitilis* showed little influence on production, although their overexpression was effective (Qiu *et al*., 2011). Moreover, complex correlations were observed among the identified exporters for primary metabolite efflux, for example, deletion of 25 potential complementary exporters in *Saccharomyces cerevisiae* could no longer block lactate efflux (Mans *et al*., 2017). These investigations all suggest more than one BGC‐independent exporter might participate in efflux of a secondary metabolite and form a compensatory transporter network to confer evolutionarily advantages for the hosts (Nigam, 2015).

The presence of BGC‐independent exporters in *Streptomyces* species might be the evolutionary event caused by horizontal gene transfer (Nigam, 2015; Jiang *et al*., 2017). The location sites of the transferred genes are uncertain, which makes BGC‐independent exporter mining challenging. Even in the well‐investigated bacteria, such as *E. coli*, the identified transporters are only the tip of the iceberg (Genee *et al*., 2016). Currently, exploration of exporters for secondary metabolites have still been a Cinderella subject in *Streptomyces*. Consequently, the limitation of available exporters hinders titer improvement through exporter engineering, especially for the hosts lacking BGC‐linked exporters. Therefore, the exporter mining strategy is quite eager to be developed for *Streptomyces* species.

Here, we focused on exploring exporters that facilitate production of macrolide biopesticides (such as avermectins and milbemycins), which are valuable in the agricultural field. In comparison to clinically used natural product medicines, agricultural natural products exhibit more urgent demands for useful genetic parts and efficient strategies for high‐yield strain constructions. For example, although milbemycin products possess high insecticidal activity and show great commercial market value, their low production restrains their applications in comparison to avermectins (Jin *et al*., 2020; Wang *et al*., 2020). *S. bingchenggensis* is a strong milbemycin producer originally found by our group (Xiang *et al*., 2007; Wang *et al*., 2010). As a result, we developed a workflow for exporter exploration and application in this species as proof of concept. The most attractive feature of this workflow is its global and systematic analytical procedure. According to genome‐wide analysis, our initial research objectives covered all exporters in a genome evolving from different ancestors and showed a wider spectrum than previous investigations focusing on BGC‐linked exporters, or the homologues of known exporters. This analytical approach provided an opportunity to clarify the effect of different types of exporters on titer improvement of desired products, which is valuable for further *Streptomyces* engineering. Moreover, this approach can also be applied to explore desired transporters for production enhancement of other secondary metabolites.

During strain engineering, the complexity of transmembrane structures of exporters probably poses a major challenge for their overexpression (Mukhopadhyay, 2015) and has been reported to be detrimental to the host cell growth (Hu *et al*., 2018; Steiger *et al*., 2019). We also observed growth inhibition when the target exporters were constitutively overexpressed by strong promoters in the present work (Fig. 3D and Fig. S6). Since essential precursors for secondary metabolite biosynthesis are all come from the cell growth process (Li *et al*., 2021), fine‐tuning the overexpression level of target exporters to alleviate growth inhibition might be important to promote final production. Previously, we developed an autoregulated fine‐tuning strategy for titer improvement of secondary metabolites using native promoters in *Streptomyces* (Li *et al*., 2018). On this basis, we combined different RBSs with native temporal promoters for more precise fine‐tuning. Here, we observed higher productions in the combinations comprising a temporal promoter with a heterogenous RBS, rather than the native one, suggesting RBS may also be as necessary as promoter when designing a high‐yield strain.

## Conclusions

Here we built a workflow to mine BGC‐independent exporters and demonstrated their applications for titer improvement of macrolide biopesticides in different *Streptomyces* producers. Our work not only identified previously unknown exporter tools for strain improvement, but also provides a useful workflow for exporter exploration, which may shed light on basic and applied investigations of exporters in *Streptomyces*.

## Experimental procedures

### Strains and cultivations

Strains used in the present work are described in Table S1. *E. coli* JM109 (Novagen) was used for all plasmids cloning procedures cultured in Luria−Bertani (LB) broth at 37°C. *E*. *coli* ET12567/pUZ8002 was used for conjugative transfer implemented in LB with 25 μg ml^‐1^ chloramphenicol and 25 μg ml^‐1^ kanamycin at 37°C. For conjugations, all *Streptomyces* strains were grown on MS agar plates (Gao *et al*., 2009). *S. bingchenggensis* and *S. cyaneogriseus* NMWT1 are deposited at China General Microbiological Culture Collection and Agricultural Research Service Culture Collection, respectively. For spore collection, *S*. *bingchenggensis*, *S*. *cyaneogriseus* NMWT1, *S*. *avermitilis* NEAU12 and their derivatives strains were grown separately at 28°C on agar plates of SKYM for 9 days (Zhang *et al*., 2016), ISP3 for 9 days (Li *et al*., 2019) and MS for 7 days (Gao *et al*., 2009). One square centimeter of spores were scraped off and then inoculated into 25 ml seed medium for mycelium growth. Seed cultures were then transferred to the 25 ml fermentation medium with 6% inoculum and shaken at 250 rpm for 9 days, 10 days and 9 days at 28°C for fermentation of *S*. *bingchenggensis*, *S*. *avermitilis* and *S*. *cyaneogriseus*, respectively. The seed and fermentation medium of *S*. *bingchenggensis*, *S*. *avermitilis* and *S*. *cyaneogriseus* were the same as previously reported (Gao *et al*., 2009; Zhang *et al*., 2016; Li *et al*., 2019).

### Construction of plasmids

Plasmids and interrelated primers used in this study are listed in Table S2 and Table S3. Plasmid pSET152T was digested by EcoRI and XbaI to generate LpSET152T used as a backbone for plasmid construction. All genes of *S*. *bingchenggensis* were amplified by using the genome of *S. bingchenggensis* BC‐101‐4 as template in the present work. To evaluate the performance of exporters, the *hrdB*p was amplified from plasmid pSET152::PhrdBmilR using primer pair PhrdB‐F/PhrdB‐R and digested with EcoRI and SpeI (Zhang *et al*., 2016). In addition, each exporter fragment was cloned from the genomic DNA of *S. bingchenggensis* by PCR using the primer pair M*_n_*‐F/M*_n_*‐R (*n* represents 1–14). The purified exporter fragments were digested with SpeI and XbaI to ligate with the double digested *hrdB*p fragment by EcoRI and SpeI and LpSET152T to generate plasmids pMiltAB1‐8 and pMilMFS1‐6, respectively. To evaluate the influence of promoters, the *kasO*p* was amplified from the plasmid pDR4‐K* using primer pair Pkaso‐F/Pkaso‐R and digested with EcoRI and SpeI (Wang *et al*., 2013). The purified exporter fragment MiltAB2 was digested with SpeI and XbaI to ligate with the double digested *kasO*p* and LpSET152T to generate plasmid kMiltAB2. The exporter fragment MiltAB2 linked with its native promoter amplified from the genome of *S. bingchenggensis* by primer pair Pnative‐F/PnM2‐R was cloned to the XbaI and EcoRI sites of LpSET152T by Gibson DNA assembly to obtain nMiltAB2. To construct the TuPPE module, the fragment P*_i_* R*_j_* was cloned from the primer pair P*_i_* R*_j_* ‐F/P*_i_* R*_j_* ‐R (*i* represents 1–5, *j* represents n, a, b, c, and d). The purified fragment P*_i_* R*_j_* was digested with EcoRI and SpeI to ligate with the SpeI/XbaI double digested corresponding exporter fragments and LpSET152T to generate plasmids pWTM*_nij_*.

### Transcriptome analysis

Cells of *S. bingchenggensis* BC‐101‐4 and ΔsbbA were harvested at 0.75, 2, 3, 4, 6, and 8 days (GEO No: GSE166795). RNA extraction, quality examination and synthesis of cDNA were described as previously (Zhang *et al*., 2016). The FPKM value of all genes from the milbemycin production stage (3–8 days) were selected for calculating the correlation between genes transcriptional level and milbemycin production. Genes with a correlation coefficient more than 0.995 and a slope more than 0.05 were chosen.

### Quantitative Real‐Time PCR (qRT‐PCR)

Cells of *S. bingchenggensis* and derivatives strains were harvested at the fourth day. Samples were frozen with liquid nitrogen and refrigerated at −80°C. After RNA extraction, quality examination, and cDNA synthesis, the qRT‐PCR experiments were implemented on a QuantStudio 5 (Applied Biosystems, Waltham, MA, USA) using PowerUp SYBR Green Master Mix (Applied Biosystems). The details of PCR procedures and reaction parameters were performed as described previously (Jin *et al*., 2020). Data of qRT‐PCR were analyzed by LightCycler®‐96 Series Software. The 16S rRNA was used as an internal control (Zhang *et al*., 2016). All experiments were run in three biological triplicates independently.

### Phylogenetic analysis

The sequences used in this work were obtained from GenBank. The neighbor‐joining algorithms was adopted to construct the phylogenetic tree by MEGA software version 6 (Saitou and Nei, 1987). The Poisson model was chosen for calculating the phylogenetic distances (Wang *et al*., 2008). Using a bootstrap procedure with 1,000 replicates to evaluate the reliability of clades. All positions containing gaps and missing data were eliminated from the dataset (complete deletion option). Tree files were viewed and embellished using online tool Interactive Tree Of Life (iTOL) v4 (Letunic and Bork, 2019).

### Promoter selection

Promoter candidates were selected based on analysis of time‐series transcriptome data of *S. bingch*enggensis BC‐101‐4 (GEO No: GSE166795). For each gene, the FPKM value of all time points (0.75, 2, 3, 4, 6, and 8 days) was normalized to that obtained at 0.75 day, resulting in a series of fold changes (|log_2_^fold change^|). After combining with milbemycin production data, hierarchical cluster analysis was performed, and promoters possessed similar patterns with milbemycin biosynthetic profile were picked out. We here chose the 500‐bp sequence upstream of the corresponding gene as the native promoter (Jin *et al*., 2020).

### Measurement of total and extracellular milbemycins

Prior to milbemycin measurement, 1.5 ml ethanol were mixed with 0.5 ml fermentation broth (9 day) of *S. bingchenggensis* and 0.5 ml supernatant of fermentation broth to extract total and extracellular milbemycins, respectively. Then the mixtures were shaken by vortex for 30 min, and the extractions containing milbemycins for measurement were collected after 15 min centrifugation at 12 000 *g*. The total and extracellular nemadectin were extracted in the same way as described above. The total and extracellular avermectin B_1a_ was extracted by using methanol instead of ethanol in the above method. Titer of milbemycins, nemadectin and avermectins were assayed by HPLC, and the detailed analytical conditions were described as previously (Jin *et al*., 2020).

### Other analytical methods

Dry cell weight was determined by collecting two‐milliliter cell cultures and drying at 55°C to a constant weight. Protein secondary structure prediction was analyzed by using the TMHMM Server v. 2.0 (http://www.cbs.dtu.dk/services/TMHMM/).

### Statistical analysis

All experiments were run in three biological triplicates independently. Data were presented as average and standard deviation. Significance was analyzed by Student’s *t*‐test, and the significance were presented as follows, ***P < 0.001, **P < 0.01, and *P < 0.05, and ‘ns’ means no significance.

## Funding Information

We gratefully acknowledge the financial support from the National Natural Science Foundation of China (31972348, 31772242 and 31672092).

## Conflict of interests

The authors declare no conflict of interest. We have filed a provisional patent for this work.

## Author contributions

S.L., X.W., and W.X. conceived and supervised the project. L.C. and S.L. designed and performed the main experiments. Z.D. and Y.Z. participated transcriptome analysis. P.J. and L.Y. participated in the fermentation experiments. L.C., S.L. and W.X. wrote and revised the manuscript.

## Supporting information


**Fig. S1**. Analysis of transcriptional levels distribution of genes in ΔsbbA.
**Fig. S2**. Transcriptional profiles of the nine selected genes in ΔsbbA.
**Fig. S3**. Protein secondary structure prediction of SBI_01019.
**Fig. S4**. Productivity comparison between BC‐101‐4 and engineered strains at different fermentation stages.
**Fig. S5**. Comparison of the transcriptional level of the corresponding genes in BC‐101‐4 and engineered strains.
**Fig. S6**. Influence of overexpressing the MiltAB2 by different promoters on cell growth.
**Fig. S7**. Transcriptional profile of genes possessed similar patterns with milbemycin biosynthesis.
**Fig. S8**. Influence of the TuPPE module containing MiltAB1 and MiltAB6 on milbemycin production.
**Fig. S9**. Titer of milbemycin A3/A4, avermectins B1a and nemadectin α in S. bingchenggensis BC‐101‐4, S. avermitilis NEAU12 and S. cyaneogriseus NMWT1.
**Fig. S10**. Comparison of yield between engineered and parent strains.
**Fig. S11**. Comparison of the dry weight between engineered and parent strains.
**Table S1**. Strains used in this work.
**Table S2**. Plasmids used in this work.
**Table S3**. Primers used in this work.
**Table S4**. The 500‐bp sequence of five native temporal promoters.
**Table S5**. Four different RBSs from commonly used cloning vectors.

**Dataset S1**. Genes correlated with the titer of milbemycin A3/A4.

**Dataset S2**. Predicted drug exporters in S. bingchenggensis.

**Dataset S3**. The major facilitator superfamily proteins and ABC superfamily proteins.

**Dataset S4**. Genes possessed similar patterns with milbemycin biosynthetic profile.
