# Supplementary material for: Mining and engineering exporters for titer improvement of macrolide biopesticides in Streptomyces
Source: Microb Biotechnol. 2021 Aug 26;15(4):1120–32. doi: 10.1111/1751-7915.13883 (PMC8966021; doi:10.1111/1751-7915.13883)
Supplement: Supplementary file 1 — Fig. S1. Analysis of transcriptional levels distribution of genes in ΔsbbA. Fig. S2. Transcriptional profiles of the nine selected genes in ΔsbbA. Fig. S3. Protein secondary structure prediction of SBI_01019. Fig. S4. Productivity comparison between BC‐101‐4 and engineered strains at different fermentation stages. Fig. S5. Comparison of the transcriptional level of the corresponding genes in BC‐101‐4 and engineered strains. Fig. S6. Influence of overexpressing the MiltAB2 by different promoters on cell growth. Fig. S7. Transcriptional profile of genes possessed similar patterns with milbemycin biosynthesis. Fig. S8. Influence of the TuPPE module containing MiltAB1 and MiltAB6 on milbemycin production. Fig. S9. Titer of milbemycin A3/A4, avermectins B1a and nemadectin α in S. bingchenggensis BC‐101‐4, S. avermitilis NEAU12 and S. cyaneogriseus NMWT1. Fig. S10. Comparison of yield between engineered and parent strains. Fig. S11. Comparison of the dry weight between engineered and parent strains. Table S1. Strains used in this work. Table S2. Plasmids used in this work. Table S3. Primers used in this work. Table S4. The 500‐bp sequence of five native temporal promoters. Table S5. Four different RBSs from commonly used cloning vectors. [file MBT2-15-1120-s004.docx]

**Supplementary files**

**Mining and engineering** **exporters for titer improvement of** **macrolide biopesticides in *Streptomyces***

*Running title: Exporter engineering in Streptomyces*

Liyang Chu^1,2,#^, Shanshan Li^2,#,*^, Zhuoxu Dong^1,2^, Yanyan Zhang^2^, Pinjiao Jin^1,2^, Lan Ye^1,2^, Xiangjing Wang^1,*^, Wensheng Xiang^1,2,*^

^1^ School of Life Science, Northeast Agricultural University, No. 59 Mucai Street, Xiangfang District, Harbin 150030, China

^2^ State Key Laboratory for Biology of Plant Diseases and Insect Pests, Institute of Plant Protection, Chinese Academy of Agricultural Sciences, Beijing 100193, China

#These authors contributed equally to this work.

*Corresponding authors: E-mail: Shanshan Li, [ssli@ippcaas.cn](mailto:ssli@ippcaas.cn); Xiangjing Wang, wangneau2013@163.com; Wensheng Xiang, [xiangwensheng@neau.edu.cn](mailto:xiangwensheng@neau.edu.cn).

# Supplementary Figures


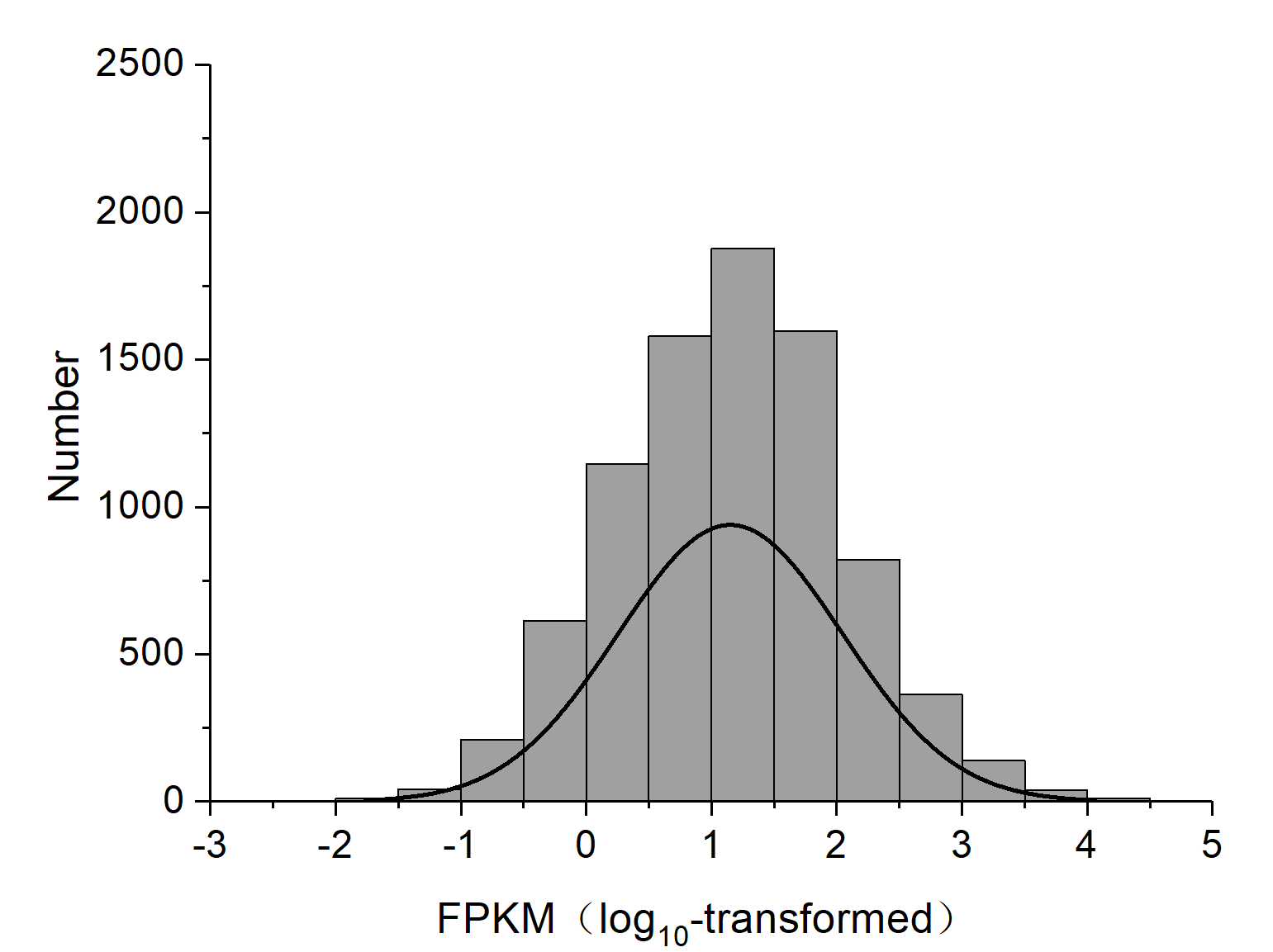


# Fig. S1 Analysis of transcriptional levels distribution of genes in ΔsbbA. FPKM values of the corresponding genes were log10 transformed. Data used were the FPKM values obtained from the eighth day.





# Fig. S2 Transcriptional profiles of the nine selected genes in ΔsbbA. Data shown were the averages and standard deviations. All data were obtained from three independent experiments.


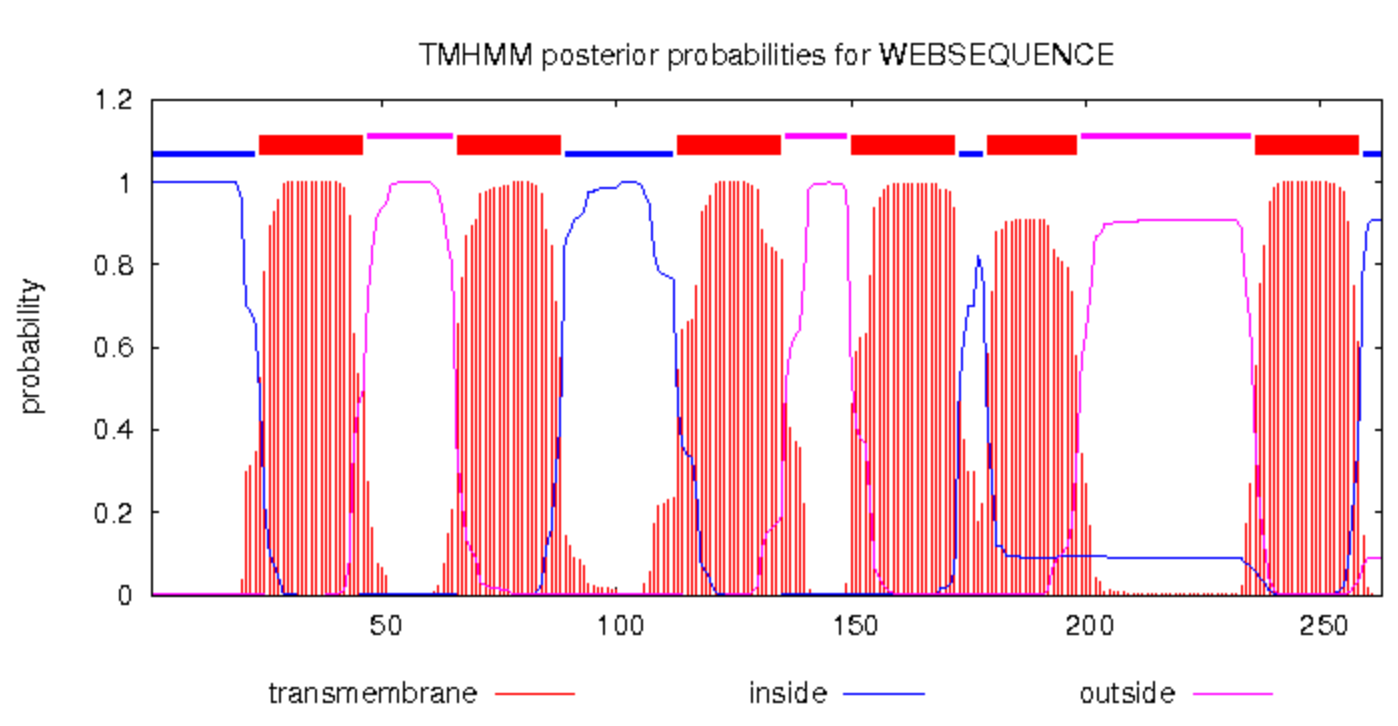


# Fig. S3 Protein secondary structure prediction of SBI_01019.





# Fig. S4 Productivity comparison between BC-101-4 and engineered strains at different fermentation stages. (A) Productivity discrepancy between BCMiltAB1 and BC-101-4. (B) Productivity discrepancy between BCMiltAB6 and BC-101-4. Data shown were the averages and standard deviations. All data were obtained from three independent experiments. Differences were analyzed by Student’s *t*-test, and *p* < 0.05 was considered statistically significant. The levels of significance are *** *p* < 0.001, ** *p* < 0.01, * *p* < 0.05, ‘ns’ means no significant difference.





# Fig. S5 Comparison of the transcription level of the corresponding genes in BC-101-4 and engineered strains. Strain names BCMiltAB2, KBCMiltAB2, and NBCMiltAB2 represent the strains overexpressing the MiltAB2 by promoter *hrdB*, *kasO*p*, and the native promoter P*_sbi_00840_* in BC-101-4, respectively. Differences were analyzed by Student’s *t*-test, and *p* < 0.05 was considered statistically significant. The levels of significance are *** *p* < 0.001, ** *p* < 0.01, * *p* < 0.05, ‘ns’ means no significant difference. Data shown were the averages and standard deviations. All data were obtained from three independent experiments.





# Fig. S6 Influence of overexpressing the MiltAB2 by different promoters on cell growth. Strain names BCMiltAB2, KBCMiltAB2, NBCMiltAB2 and WMT22d represent the strains overexpressing the MiltAB2 by promoter *hrdB*, *kasO*p*, native promoter P*_sbi_00840_* and P2 (linked with Rd) in BC-101-4, respectively. The data of BC-101-4 was set as control. All data were obtained from three independent experiments. Data shown were the averages and standard deviations. Differences were analyzed by Student’s *t*-test, the levels of significance are *** *p* < 0.001, ** *p* < 0.01, * *p* < 0.05, ‘ns’ means no significant difference.


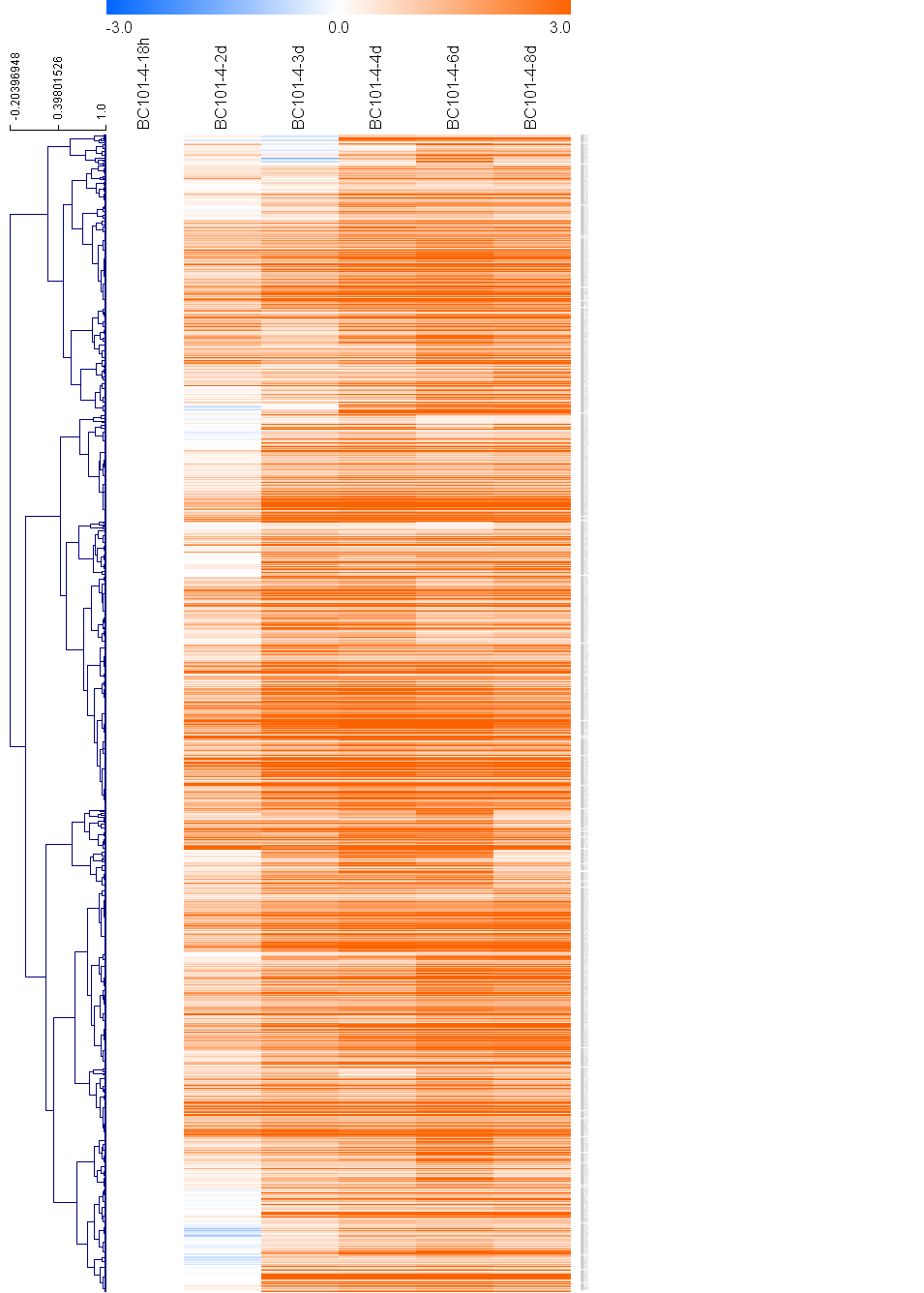


# Fig. S7 Transcriptional profile of genes possessed similar patterns with milbemycin biosynthesis. Data shown were the averages and standard deviations. All data were obtained from three independent experiments.


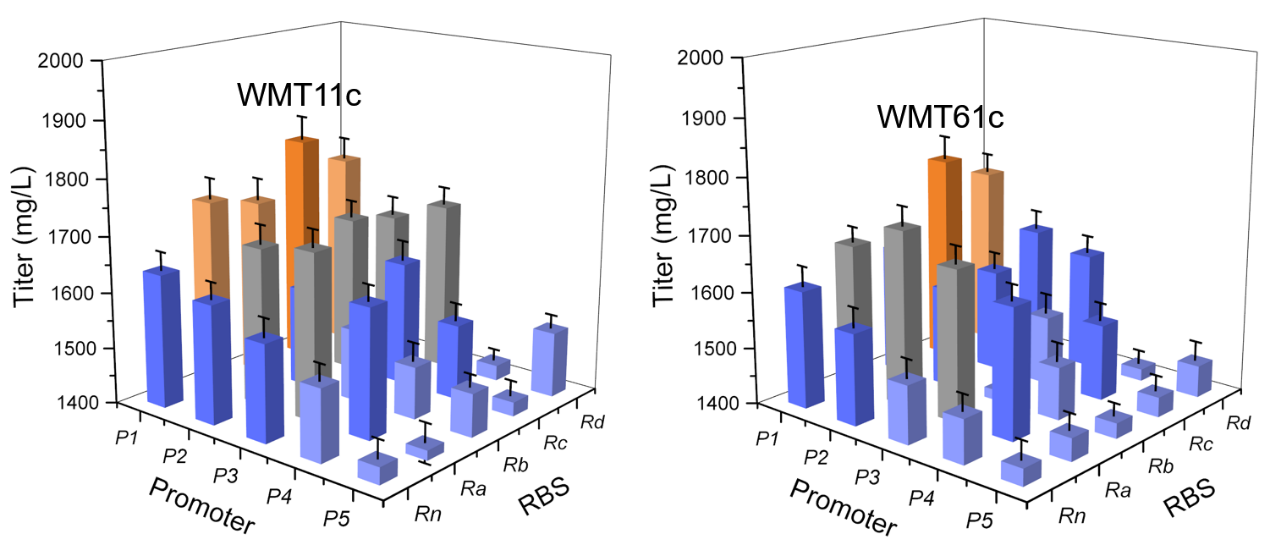


# Fig. S8 Influence of the TuPPE module containing MiltAB1 and MiltAB6 on milbemycin production. Data shown were the averages and standard deviations. All data were obtained from three independent experiments.





# Fig. S9 Titer of milbemycin A3/A4, avermectins B_1a_ and nemadectin α in *S. bingchenggensis* BC-101-4, *S.* *avermitilis* NEAU12 and *S. cyaneogriseus* NMWT1. Data shown were the averages and standard deviations. All data were obtained from three independent experiments.





# Fig. S10 Comparison of yield between engineered and parent strains. (A) Comparison of yield between engineered *S. avermitilis* and their parent strain. (B) Comparison of yield between engineered *S. cyaneogriseus* and their parent strain. The gray and orange columns indicate the parent and engineered strains, respectively. Strain names containing MT11c, MT22d and MT61c represent the strains integrated with the MiltAB1, MiltAB2 and MiltAB6 involved TuPPE in corresponding *Streptomyces* host. Data shown were the averages and standard deviations. All data were obtained from three independent experiments. Differences were analyzed by Student’s *t*-test, and *p* < 0.05 was considered statistically significant. The levels of significance are *** *p* < 0.001, ** *p* < 0.01, * *p* < 0.05, ‘ns’ means no significant difference.





# Fig. S11 Comparison of the dry weight between engineered and parent strains. (A) Comparison of the dry weight between engineered *S. avermitilis* and their parent strain. (B) Comparison of the dry weight between engineered *S. cyaneogriseus* and their parent strain. The gray and orange columns indicate the dry weight of the parent and engineered strains in different time points. Strain names containing MH1, MH2 and MH6 represent the strains integrated with the MiltAB1, MiltAB2 and MiltAB6 controlled by promoter *hrdB* in corresponding host. Strain names containing MT11c, MT22d and MT61c represent the strains integrated with the MiltAB1, MiltAB2 and MiltAB6 involved TuPPE module in corresponding host. Data shown were the averages and standard deviations. All data were obtained from three independent experiments. Differences were analyzed by Student’s *t*-test, and *p* < 0.05 was considered statistically significant. The levels of significance are *** *p* < 0.001, ** *p* < 0.01, * *p* < 0.05, ‘ns’ means no significant difference.

# Supplementary Tables

# Table S1 Strains used in this work

| Name | Description | Source | |
| --- | --- | --- | --- |
| *Escherichia coli* |  | |  |
| *E. coli* JM109 | Host for all plasmids cloning procedures | | Novagen |
| ET12567 (pUZ8002) | Donor strain for conjugation between *E. coli* and *Streptomyces* | | (Kieser*, et al.*, 2000) |
| *S.* *bingchenggensis* |  | |  |
| BC-101-4 | Milbemycin producer | | (Wang*, et al.*, 2009) |
| ΔsbbA | A high-yielding milbemycin producer | | (He*, et al.*, 2018) |
| BC-101-4C | BC-101-4 integrated with the control plasmid (pSET152T) | | This work |
| KBCMiltAB2 | Overexpressing the MiltAB2 by promoter *kasO*p* in BC-101-4. | | This work |
| NBCMiltAB2 | Overexpressing the MiltAB2 by its native promoter in BC-101-4. | | This work |
| BCMiltAB*_n_* | Overexpressing the MiltAB*_n_* by promoter *hrdB* in BC-101-4. MiltAB*_n_* indicates MiltAB1, SBI_01019−SBI_01020; MiltAB2， SBI_00839−SBI_00840; MiltAB3, SBI_07695−SBI_07696; MiltAB4, SBI_04806−SBI_04807; MiltAB5, SBI_05053−SBI_05054; MiltAB6, SBI_05114−SBI_05116; MiltAB7, SBI_08914−SBI_08915; MiltAB8, SBI_09667−SBI_09668, respectively | | This work |
| BCMilMFS*_n_* | Overexpressing MFS exporters by promoter *hrdB* in BC-101-4. MilMFS*_n_* indicates MilMFS1, SBI_02333; MilMFS2, SBI_03552; MilMFS3, SBI_05341; MilMFS4, SBI_08594; MilMFS5, SBI_00964; MilMFS6, SBI_07918, respectively | | This work |
| WTM1*_ij_* | Overexpressing the MiltAB1 by promoter P*_i_* and RBS R*_j_* in BC-101-4. P*_i_* indicates P1, the promoter region of *sbi_04846*; P2, the promoter region of *sbi_09292*; P3, the promoter region of *sbi_03281*; P4, the promoter region of *sbi_03944*; P5, the promoter region of *sbi_05812*, respectively, R*_j_* indicates Rn, the native RBS of P*_i_*; Ra, the RBS of resistance gene (apr) from the pSET152; Rb, the RBS of *sfgfp* from the pCumate; Rc, the RBS of resistance gene (kan) from the pDR41; Rd, the RBS of *otrR* from the pIJ-Potr, respectively | | This work |
| WTM2*_ij_* | Overexpressing the MiltAB2 by promoter P*_i_* and RBS R*_j_* in BC-101-4. P*_i_* indicates P1, the promoter region of *sbi_04846*; P2, the promoter region of *sbi_09292*; P3, the promoter region of *sbi_03281*; P4, the promoter region of *sbi_03944*; P5, the promoter region of *sbi_05812*, respectively, R*_j_* indicates Rn, the native RBS of P*_i_*; Ra, the RBS of resistance gene (apr) from the pSET152; Rb, the RBS of *sfgfp* from the pCumate; Rc, the RBS of resistance gene (kan) from the pDR41; Rd, the RBS of *otrR* from the pIJ-Potr, respectively | | This work |
| WTM6*_ij_* | Overexpressing the MiltAB6 by promoter P*_i_* and RBS R*_j_* in BC-101-4. P*_i_* indicates P1, the promoter region of *sbi_04846*; P2, the promoter region of *sbi_09292*; P3, the promoter region of *sbi_03281*; P4, the promoter region of *sbi_03944*; P5, the promoter region of *sbi_05812*, respectively, R*_j_* indicates Rn, the native RBS of P*_i_*; Ra, the RBS of resistance gene (apr) from the pSET152; Rb, the RBS of *sfgfp* from the pCumate; Rc, the RBS of resistance gene (kan) from the pDR41; Rd, the RBS of *otrR* from the pIJ-Potr, respectively | | This work |
| BC04 | Industrial milbemycin producer | | (Zhang*, et al.*, 2016) |
| HTM11c | Overexpressing the MiltAB1 by promoter P1 and RBS Rc in BC04 | | This work |
| HTM22d | Overexpressing the MiltAB2 by promoter P2 and RBS Rd in BC04 | | This work |
| HTM61c | Overexpressing the MiltAB6 by promoter P1 and RBS Rc in BC04 | | This work |
| *S.* *avermitilis* |  | |  |
| NEAU12 | Industrial avermectin producer | | (Jin*, et al.*, 2020) |
| NEAU12C | NEAU12 integrated with the control plasmid (pSET152T) | | This work |
| SMH1 | Overexpressing the MiltAB1 by promoter *hrdB* in NEAU12 | | This work |
| SMH2 | Overexpressing the MiltAB2 by promoter *hrdB* in NEAU12 | | This work |
| SMH6 | Overexpressing the MiltAB6 by promoter *hrdB* in NEAU12 | | This work |
| SMT11c | Overexpressing the MiltAB1 by promoter P1 and RBS Rc in NEAU12 | | This work |
| SMT22d | Overexpressing the MiltAB2 by promoter P2 and RBS Rd in NEAU12 | | This work |
| SMT61c | Overexpressing the MiltAB6 by promoter P1 and RBS Rc in NEAU12 | | This work |
| *S. cyaneogriseus* |  | |  |
| NMWT1 | Nemadectin producer | | (Li*, et al.*, 2019) |
| NMWT1C | NMWT1 integrated with the control plasmid (pSET152T) | | This work |
| NMH1 | Overexpressing the MiltAB1 by promoter *hrdB* in NMWT1 | | This work |
| NMH2 | Overexpressing the MiltAB2 by promoter *hrdB* in NMWT1 | | This work |
| NMH6 | Overexpressing the MiltAB6 by promoter *hrdB* in NMWT1 | | This work |
| NMT11c | Overexpressing the MiltAB1 by promoter P1 and RBS Rc in NMWT1 | | This work |
| NMT22d | Overexpressing the MiltAB2 by promoter P2 and RBS Rd in NMWT1 | | This work |
| NMT61c | Overexpressing the MiltAB6 by promoter P1 and RBS Rc in NMWT1 | | This work |

# Table S2 Plasmids used in this work

| Name | Description | Source |
| --- | --- | --- |
| pSET152 | Am^r^, integrative *E. coli-Streptomyces* shuttle vector | (Kieser, *et al.*, 2000) |
| pSET152T | Am^r^, derived from pSET152, containing a terminator | Laboratory stock |
| kMiltAB2 | Am^r^, derived from pSET152T, containing the MiltAB2 driven by promoter *kasO*p* | This work |
| nMiltAB2 | Am^r^, derived from pSET152T, containing the MiltAB2 driven by its native promoter | This work |
| pMiltAB*_n_* | Am^r^, derived from pSET152T, containing the MiltAB*_n_* driven by promoter *hrdB*, MiltAB*_n_* indicates MiltAB1, SBI_01019−SBI_01020; MiltAB2， SBI_00839−SBI_00840; MiltAB3, SBI_07695−SBI_07696; MiltAB4, SBI_04806−SBI_04807; MiltAB5, SBI_05053−SBI_05054; MiltAB6, SBI_05114−SBI_05116; MiltAB7, SBI_08914−SBI_08915; MiltAB8, SBI_09667−SBI_09668, respectively | This work |
| pMilMFS*_n_* | Am^r^, derived from pSET152T, containing the MFS exporters driven by promoter *hrdB*, MilMFS*_n_* indicates MilMFS*_n_* indicates MilMFS1, SBI_02333; MilMFS2, SBI_03552; MilMFS3, SBI_05341; MilMFS4, SBI_08594; MilMFS5, SBI_00964; MilMFS6, SBI_07918, respectively | This work |
| pMiltAB*_nij_* | Am^r^, derived from pSET152T, containing the MiltAB*_n_* driven by promoter P*_i_* and RBS R*_j_*. MiltAB*_n_* indicates MiltAB1, SBI_01019−SBI_01020; MiltAB2, SBI_00839−SBI_00840; MiltAB6, SBI_05114−SBI_05116, respectively, P*_i_* indicates P1, the promoter region of *sbi_04846*; P2, the promoter region of *sbi_09292*; P3, the promoter region of *sbi_03281*; P4, the promoter region of *sbi_03944*; P5, the promoter region of *sbi_05812*, respectively, R*_j_* indicates Rn, the native RBS of P*_i_*; Ra, the RBS of resistance gene (apr) from the pSET152; Rb, the RBS of *sfgfp* from the pCumate; Rc, the RBS of resistance gene (kan) from the pDR41; Rd, the RBS of *otrR* from the pIJ-Potr, respectively | This work |

# Table S3 Primers used in this work

| Primers | Sequence (5'-3')^a^ | Usage |
| --- | --- | --- |
| Q16s-F | TGTCGTGAGATGTTGGGTTAAG | For qRT-PCR of 16s |
| Q16s-R | TCATTGTACCGGCCATTGTAG |  |
| Q839-F | ATCGCCTCGTACACCTACA | For qRT-PCR of *sbi*_*00839* |
| Q839-R | AACTGCGCGAAGGACAC |  |
| Q840-F | GTCGCTCCTACGCCAAG | For qRT-PCR of *sbi*_*00840* |
| Q840-R | CGCCATCAGGTGGTTGAA |  |
| PhrdB-F | CG**GAATTC**TCTAGACCGCCTTCCGCCG | For amplification of the promoter *hrdB* |
| PhrdB-R | GG**ACTAGT**GAACAACCTCTCGGAACGTTGAAAA |  |
| Pkaso-F | CG**GAATTC**TGTTCACATTCGAACGGTCTC | For amplification of the promoter *kasO*p* |
| Pkaso-R | GG**ACTAGT**AACTCCCCCAGTCCTGCA |  |
| Pnative-F | *AGGAAACAGCTATGACATGATTAC****GAATTC***GTCGACGCTGGCGTTGTCCGCC | For amplification of genes *sbi*_*00839*-*sbi*_*00840* and its native promoter fragment |
| PnM2-R | *GGAAAGACGACAAAACTTTAGATC****TCTAGA***TCAGGCGTCGGTCCGCACGA |  |
| M1-F | GG**ACTAGT**GTGATCGAAGTGCGCGACCTCA | For amplification of genes *sbi*_*01019*-*sbi*_*01020* |
| M1-R | GC**TCTAGAGGATCC**TCAAGGGTCGCGGCGGCGCAG |  |
| M2-F | GG**ACTAGT**ATGATCGAAGCTCGTGAGCTG | For amplification of genes *sbi*_*00839*-*sbi*_*00840* |
| M2-R | GC**TCTAGAGGATCC**TCAGGCGTCGGTCCGCACGA |  |
| M3-F | GG**ACTAGT**ATGACGCGATACGAACACA | For amplification of genes *sbi*_*07695*-*sbi*_*07696* |
| M3-R | GC**TCTAGAGGATCC**TCAGGTCTTGTTGCGGAACTTC |  |
| M4-F | GG**ACTAGT**GTGGTGACGATTCACCAGCT | For amplification of genes *sbi*_*04806*-*sbi*_*04807* |
| M4-R | GC**TCTAGAGGATCC**TCAGGCCGTGACATCGCGGCGCG |  |
| M5-F | GG**ACTAGT**TTGATCGTCGTGCTGCAACTGGTGC | For amplification of genes *sbi*_*05053*-*sbi*_*05054* |
| M5-R | GC**TCTAGAGGATCC**TCACTCCGCCTCGGCCAC |  |
| M6-F | GG**ACTAGT**GTGAGCGACCCAGGGATCGTCG | For amplification of genes *sbi*_*05114*-*sbi*_*05116* |
| M6-R | GC**TCTAGAGGATCC**CTAGGTGTCGCGCATGCGCAGCAG |  |
| M7-F | GG**ACTAGT**GTGCTGGCCCAGGCGCTCGTA | For amplification of genes *sbi*_*08914*-*sbi*_*08915* |
| M7-R | GC**TCTAGAGGATCC**TCAGGTGCGGCCGTGCCAGGACGC |  |
| M8-F | GG**ACTAGT**ATGGAAGAACTCCGCGCAATCCG | For amplification of genes *sbi*_*09667*-*sbi*_*09668* |
| M8-R | GC**TCTAGAGGATCC**TTATCCCTTCGGGCTGGTG |  |
| M9-F | GG**ACTAGT**ATGGACAGCGATACGGACGCGAA | For amplification of genes *sbi*_*02333* |
| M9-R | GC**TCTAGAGGATCC**TCAGTGACGCGTCGCGCT |  |
| M10-F | GG**ACTAGT**ATGGCCCAGCCCCTCGACAC | For amplification of genes *sbi*_*03552* |
| M10-R | GC**TCTAGAGGATCC**CTAGCCCAGGTGCACCGGAAG |  |
| M11-F | GG**ACTAGT**ATGCCCCTCGCCCTGCTGGC | For amplification of gene *sbi*_*05341* |
| M11-R | GC**TCTAGAGGATCC**TCAGCGTACGGCTGTCGCGTCCT |  |
| M12-F | GG**ACTAGT**GTGAACGTCACACCATCGGCGA | For amplification of gene *sbi*_*08594* |
| M12-R | GC**TCTAGAGGATCC**CTACGTCTGCGTTCGGCCC |  |
| M13-F | GG**ACTAGT**ATGTCCGCCACCGCCACCGCC | For amplification of gene *sbi*_*00964* |
| M13-R | GC**TCTAGAGGATCC**CTATCGCTCGCCGTCCCGGAAC |  |
| M14-F | GG**ACTAGT**ATGACCGGCGACCCGGGGG | For amplification of gene *sbi*_*07918* |
| M14-R | GC**TCTAGAGGATCC**TCAGTCCGCCGTCGCCGGG |  |
| P1R*_j_*-F | CG**GAATTC**CACAAGTAAGCGGCGTGGTGTTC | For amplification of the fragments P1R*_j_* |
| P1R1-R | GG**ACTAGT**GCGGTGGCTTCTCTCGTGAGCAGTGCGTGAGAGTTAC | For amplification of the fragment P1R1 |
| P1Ra-R | GG**ACTAGT**GCGGTGGCTCTCCGTGAGCAGTGCGTGAGAGTTAC | For amplification of the fragment P1Ra |
| P1Rb-R | GG**ACTAGT**GCGGTGGCTCTTTCGTGAGCAGTGCGTGAGAGTTAC | For amplification of the fragment P1Rb |
| P1Rc-R | GG**ACTAGT**GCGGTGGCTTCCTCGTGAGCAGTGCGTGAGAGTTAC | For amplification of the fragment P1Rc |
| P1Rd-R | GG**ACTAGT**GCGGTGGCTCTCCCCGTGAGCAGTGCGTGAGAGTTAC | For amplification of the fragment P1Rd |
| P2R*_j_*-F | CG**GAATTC**CGACCGAGCCGCCGACTGAGCCGC | For amplification of the fragments P2R*_j_* |
| P2R2-R | GG**ACTAGT**GCGCGCCGCTCTCCCATGTCTTCCATATTTTCCCTGC | For amplification of the fragment P2R2 |
| P2Ra-R | GG**ACTAGT**GCGCGCCGCCTCCATGTCTTCCATATTTTCCCTGC | For amplification of the fragment P2Ra |
| P2Rb-R | GG**ACTAGT**GCGCGCCGCCTTTCATGTCTTCCATATTTTCCCTGC | For amplification of the fragment P2Rb |
| P2Rc-R | GG**ACTAGT**GCGCGCCGCTCCTCATGTCTTCCATATTTTCCCTGC | For amplification of the fragment P2Rc |
| P2Rd-R | GG**ACTAGT**GCGCGCCGCCTCCCCATGTCTTCCATATTTTCCCTGC | For amplification of the fragment P2Rd |
| P3R*_j_*-F | CG**GAATTC**GGTCGTAGCGAACGGTGGCGAT | For amplification of the fragments P3R*_j_* |
| P3R3-R | GG**ACTAGT**GGTGTTGCCCTTCAGGTGCGGGGTTGCCGTTGT | For amplification of the fragment P3R3 |
| P3Ra-R | GG**ACTAGT**GGTGTTGCCCTCCAGGTGCGGGGTTGCCGTTGT | For amplification of the fragment P3Ra |
| P3Rb-R | GG**ACTAGT**GGTGTTGCCCTTTCAGGTGCGGGGTTGCCGTTGT | For amplification of the fragment P3Rb |
| P3Rc-R | GG**ACTAGT**GGTGTTGCCTCCTCAGGTGCGGGGTTGCCGTTGT | For amplification of the fragment P3Rc |
| P3Rd-R | GG**ACTAGT**GGTGTTGCCCTCCCCAGGTGCGGGGTTGCCGTTGT | For amplification of the fragment P3Rd |
| P4R*_j_*-F | CG**GAATTC**GCCGCGGGGTGACCTGGCGGA | For amplification of the fragments P4R*_j_* |
| P4R4-R | GG**ACTAGT**GAAAGCCCCCTTGGGGTGAATGGAGGCGCGAC | For amplification of the fragment P4R4 |
| P4Ra-R | GG**ACTAGT**GAAAGCCTCCGGGGTGAATGGAGGCGCGAC | For amplification of the fragment P4Ra |
| P4Rb-R | GG**ACTAGT**GAAAGCCTTTCGGGGTGAATGGAGGCGCGAC | For amplification of the fragment P4Rb |
| P4Rc-R | GG**ACTAGT**GAAAGCTCCTCGGGGTGAATGGAGGCGCGAC | For amplification of the fragment P4Rc |
| P4Rd-R | GG**ACTAGT**GAAAGCCTCCCCGGGGTGAATGGAGGCGCGAC | For amplification of the fragment P4Rd |
| P5R*_j_*-F | CG**GAATTC**CATCGGGGAGCTGCGCGCCGACG | For amplification of the fragments P5R*_j_* |
| P5R5-R | GG**ACTAGT**GCTTCTGCCGCCTCCTGCGTACGGCGCGTACGACGC | For amplification of the fragment P5R5 |
| P5Ra-R | GG**ACTAGT**GCTTCTGCCGCTCCGCGTACGGCGCGTACGACGC | For amplification of the fragment P5Ra |
| P5Rb-R | GG**ACTAGT**GCTTCTGCCGCTTTCGCGTACGGCGCGTACGACGC | For amplification of the fragment P5Rb |
| P5Rc-R | GG**ACTAGT**GCTTCTGCCGTCCTCGCGTACGGCGCGTACGACGC | For amplification of the fragment P5Rc |
| P5Rd-R | GG**ACTAGT**GCTTCTGCCGCTCCCCGCGTACGGCGCGTACGACGC | For amplification of the fragment P5Rd |

^a^ Bold type characters indicate restriction enzyme sites, italic characters indicate the homologous fragments, and the underline characters indicate reverse complementary sequence of the RBSs.

# Table S4 The 500-bp sequence of five native temporal promoters

| Promoters | Sequence (5'-3')^a^ |
| --- | --- |
| P*_sbi_04846_* (P1) | CACAAGTAAGCGGCGTGGTGTTCTAATTCGGCGAGGTCCAGGAACGGTGCGGGGAGGGTGCCTCGGTAGTCCTCCCACCATCCTTGGCCGCGCTGTTCGTACGTCATGGCCGCCAGTGAGTCGATGAGCGCGGTGTCGGCGCACTTGTAGAACGCCGCGAGGCGGCGCACGCGCTCCTCGCTGATCCCATAGCGGCCCGTCTCGATGTGGCTGATCTGAGGTTGCCCGCCTCCGAGCAGGGCGCCCGCTTCACGGGCGGGCATGCCCGCCGCCTCGCGCATCTTGCGCAGCTCAACTCCCAGGCGCACCTGGCGTGCTGTGGGGTTACTCCTCGGCGGCATCGAGCCCTTCTCCCGGTTTGCTCATGAGTGTGCCGCTCCACCCTGCCACGGTCCACACTCGGTGTGATGACTTTCGCGGACATGGTTGCGACGGTGCATGCATGGCCTCTACGGTCTGTGATGTAACTCTCACGCACTGCTCACGAGAGAAGCCACCGC |
| P*_sbi_09292_* (P2) | CGACCGAGCCGCCGACTGAGCCGCCGACTGAGCACGGGCGCAGGTCGGCGGAGTCTTCCTGAGTCGCACCGGGTCGCCCTGAGTCGTACCGGGTCGCGCTTGTCGAGACAGCGCGAGGAGCACTTTTCGGGACACCAGTCGCCAGGGTCGCACAGGAGCGCGACCGCGCTCCCCGTGCGCCCCCAGTGACCCCCCGGCGCCCCGCTCCGCGACGGCCTTCAACGCGCCCCACGACCTGATGCCCGTGGAGCCGCCCGCCTCGCACCCCATGGCGCACTCCAGGGCGTCGCATGGCCCACTCCAGGGCGCGGTGCCGACGGTCTCATCGGCGGCGCTCACCGCGCTCTCCACGCGCCGCCCCTTGCCTCTTGTGGCGATCCAGTGGTGAACTTGGACCTTTTCGGATCATAAGTGGGCACCAGTGCGCCCTGGCATCCCGTGCCTGTCCCGGGCACACTGCGCAGCAGGGAAAATATGGAAGACATGGGAGAGCGGCGCGC |
| P*_sbi_03281_* (P3) | GGTCGTAGCGAACGGTGGCGATGAACTCGTCCCCGACCGTGGCGGCCAGGCCCACCCGGTCGACGTAGTCATGGTGCGTGAAGCGGTGCACGTCGCGGTCGGAGAGCCGGGGGTAGGGCGCGAAGAAGCGGTAGTACTTGGACTCGTCCGAGACCCGCTCGTAGAAGCTGACCAGGCGCTGCGCGTCGTCAGCGGTGATGGGGCGGATCCGGGCGGTGCCGCCGTCGCGGAGCACCACATCGGCCTCCCAGTGGGCCGGGTAGGCGTGGGTGTCGGACGGCTGCCGCATGCGGCCAAGACTACGGCCCGGGCGGACATGGGTCCGGGCGTCCGAATGGCGGACACCCTTGCGTTGCGGGGAGGTGTCGAGGGAAAGTCCATAATCGGACCTGTACGGGCACGGCCGGGCCTACTCGGACATCGTTCCGGACAGACCCAGACATGCACCCCGTATGATATTGGTCTAGACAACGGCAACCCCGCACCTGAAGGGCAACACC |
| P*_sbi_03944_* (P4) | GCCGCGGGGTGACCTGGCGGAGCTGGCGGGGAAGCGGATGTTCCTGACGAGCTTCCAGCAGTCGTTTCCGCGACCGGAACTCATCTATGGGGACCATGAGTTCCGGCGGGCGGAGAAGGACGACACCTATCAGCGCGTCACGTACGACCACGAGCTGCTGACCATCGGGGGCGTCCCCGCGTGGCTGCTGACCGTGGGCGGCGGCCTGCTGGCCGTGCCGGCCGCCGCGCTCACGCTGCTGATGGCGGCCAGGCGGCGCAGGCGGGCGGCGCGTTTCTGGGGGCCGGGAGGCCCGCCCGGGCCGGGGGACTCACCGGGGTATCCGCAGCACCTGTCGCCGGGTCAGGACAGCTTGCCGTCGTAGTGCGGGGGCGTTGTTCATGACGCCACCGCACATACGCCAACGACACTTGCCCGCCCGTTCGTTGACTGGTGGGCGGATAGATGGGTATGTCATGTACTGACGGGGTCGCGCCTCCATTCACCCCAAGGGGGCTTTC |
| P*_sbi_05812_* (P5) | CATCGGGGAGCTGCGCGCCGACGGCCTGCCGACCGGTGCGGTCATCATCAACATGGTGCGACCGGCGATCCTCGACCACGCGGCCGTCGACGCCGCCGCCAACGGCCGGCGCGCCGCCGTCGCCAAGGCCCTGTCCCAGGCGGGGCTGGGCGGTGCGCGCCGCGGTGGGCTCGCCGAGCGGCTGGTGGACCCGCTGCTGGAGCAGGCCCGTGAGCACGCGGAGCGGGTCGCCCTGGAGCGGGCGCAGCGGGCGGAGCTGACGGGCCTGGAGCTGCCCCTGTACGAGCTGGAGCTGCTCACCGACGGCGTCGACCTGGCCGGCCTGTACCGGCTCGCTACGGATCTGCGTAAGCAGTGGCCGATGTGAGCCGCACGGGCGACGGCGGCGACACGAGCGGCGCGACACGAGCGGCGCAATCCACACCATCAGCGCAATCGGCATGAACGGTACGAGCGGGCGGTACGCGTCGTACGCGCCGTACGCAGGAGGCGGCAGAAGC |

^a^ The underline characters indicate the predicted RBS of the promoters.

# Table S5 Four different RBSs from commonly used cloning vectors

| Name | Description | Sequence (5'-3') |
| --- | --- | --- |
| Ra | The RBS of resistance gene (apr) from the pSET152 | GGAG |
| Rb | The RBS of *sfgfp* from the pCumate | GAAAG |
| Rc | The RBS of resistance gene (kan) from the pDR41 | GAGGA |
| Rd | The RBS of *otrR* from the pIJ-Potr | GGGGAG |

**Supplementary Datasets**

# Dataset S1 Genes correlated with the titer of milbemycin A3/A4

# Dataset S2 Predicted drug exporters in *S. bingchenggensis*

# Dataset S3 The major facilitator superfamily proteins and ABC superfamily proteins

# Dataset S4 Genes possessed similar patterns with milbemycin biosynthetic profile

# References

He, H., Ye, L., Li, C., Wang, H., Guo, X., Wang, X., *et al*. (2018) SbbR/SbbA, an important ArpA/AfsA-like system, regulates milbemycin production in *Streptomyces bingchenggensis*, *Front Microbiol* **9**: 1064.

Jin, P., Li, S., Zhang, Y., Chu, L., He, H., Dong, Z., and Xiang, W. (2020) Mining and fine-tuning sugar uptake system for titer improvement of milbemycins in *Streptomyces bingchenggensis*, *Synth Syst Biotechnol* **5**: 214-221.

Kieser, T., Bibb, M.J., Buttner, M.J., Chater, K.F., Hopwood, D.A., Charter, K., *et al*. (2000) Practical *Streptomyces* Genetics, Norwich: The John Innes Foundation, pp. 613.

Li, C., He, H., Wang, J., Liu, H., Wang, H., Zhu, Y., *et al*. (2019) Characterization of a LAL-type regulator NemR in nemadectin biosynthesis and its application for increasing nemadectin production in *Streptomyces cyaneogriseus*, *Sci China Life Sci* **62**: 394-405.

Wang, X.J., Wang, X.C., Xiang, W.S. (2009) Improvement of milbemycin-producing *Streptomyces bingchenggensis* by rational screening of ultraviolet- and chemically induced mutants, *World J Microbiol Biotechnol* **25**: 1051-1056.

Zhang, Y., He, H., Liu, H., Wang, H., Wang, X., and Xiang, W. (2016) Characterization of a pathway-specific activator of milbemycin biosynthesis and improved milbemycin production by its overexpression in *Streptomyces bingchenggensis*, *Microb Cell Fact* **15**: 152.
